# Impact of chlorhexidine digluconate and temperature on curli production in *Escherichia coli*—consequence on its adhesion ability

**DOI:** 10.3934/microbiol.2017.4.915

**Published:** 2017-12-01

**Authors:** Laurent Coquet, Antoine Obry, Nabil Borghol, Julie Hardouin, Laurence Mora, Ali Othmane, Thierry Jouenne

**Affiliations:** 1 CNRS UMR6270, Normandie University, UNIROUEN, Plate-forme PISSARO, Mont-Saint-Aignan, France; 2 INSERM U905, Normandie University, UNIROUEN, Plate-forme PISSARO, France; 3 Biophysical Laboratory, Faculty of Medicine of Monastir, Monastir, Tunisia; 4 INSERM U1148, Laboratory for Vascular Transitional Sciences, Galileo Institute, Paris 13 University, Sorbonne Paris Cité, Villetaneuse, France

**Keywords:** biofilm, chlorhexidine, curli, *Escherichia coli*, outer membrane, proteome, Cpx-TCS

## Abstract

Chlorhexidine-Digluconate (CHX-Dg) is a biocide widely used as disinfectant or antiseptic in clinical and domestic fields. It is often found in the formulation of solutions to treat superficial wounds. Nevertheless, few studies have focused on its effects on *Escherichia coli* while this bacterium is commonly involved in mixed infections. Therefore, the impact of CHX-Dg and temperature on *E. coli* was investigated; particularly the curli production. In accordance with bibliographic data, the curli production decreased when the temperature of the culture was shift from 30 °C to 37 °C. The bacterial adhesion to abiotic surfaces was also reduced. Surprisingly, the curli production at 37 °C was maintained in presence of antiseptic and the bacterial adhesion was improved at a very low concentration (1 µg ml^−1^) of CHX-Dg. Complementary investigations with a *cpxR* mutant demonstrated that the CpxA/R-TCS (Two-Component System) is involved in the temperature-dependent control of the curli expression. Indeed, the curli production was not altered by the growth temperature in the mutant. Otherwise, no relationship between CHX-Dg and the Cpx-TCS was shown. A subsequent proteomic investigation revealed the alteration of the production of 44 periplasmic and outer membrane proteins in presence of CHX-Dg. These proteins are involved in the transport of small molecules, the envelope integrity, the stress response as well as the protein folding.

## 1. Introduction

Chlorhexidine digluconate (CHX-Dg) is an antiseptic used for over 50 years. It is particularly used in dentistry medicine. Nevertheless, CHX-Dg is commonly found in the formulation of disinfectants and antiseptics due to its broad spectrum activity. It is mainly used to decontaminate the medical materials and the skin to prevent the surgical infections. CHX-Dg is also found in the composition of ophthalmic solutions to preserve the contact lens [1],[2]. Its mode of action results from its di-cationic structure. Its positive charges interact with the negatively charged groups of the bacterial cell wall, causing widespread membrane damage and leakage of cellular content.

The ability of bacteria to attach to abiotic surfaces and the high resistance of sessile bacteria to biocides are the major causes of concern for many industries, including the medical and food industries [3]. A large number of factors, e.g., hydrophobicity, surface charge, surface structures including curli and outer membrane proteins (OMPs), play a role in the bacteria attachment to surfaces [4]. It is the reason why we focused here on these bacterial proteinaceous determinants.

Curli, discovered in the late 1980s, are major proteinaceous components of cell surfaces in many *Enterobacteriaceae*. Their beta-sheet rich fold confers structurally and biochemically properties close to the amyloid fibers, responsible of human neurodegenerative diseases such as Alzheimer's, Huntington's and prion diseases. Like amyloid fibers, the curli present a remarkably stability by a high resistance to thermic, chemical and enzymatic denaturation. The Congo Red (CR) assay is a simple method and convenient way to estimate the curli production [5]. Thus, the observation of colony morphotypes after growth on CR agar plates can easily highlight the production of curli and/or cellulose [6].

Curli expression depends on different environmental and physiological factors. The growth temperature is the main parameter known to modulate the curli gene expression. In most *E. coli* laboratory strains, including the K-12 strain, the curli expression is high below 30 °C and absent at 37 °C [7]. Other environmental parameters influence the curli expression, e.g. osmolarity or nutrient limitation [8],[9]. The genes involved in curli production are clustered in the *csgBAC* and *csgDEFG* operons. *CsgD* encodes a key regulator that positively regulates the production of curli and cellulose [10]. Curli operons are controlled by many direct regulators: transcriptional regulators such as RpoS (positive or negative regulations), TCSs (Two-Component Systems) such as EnvZ/OmpR (positive regulation) and CpxA/R (negative regulation), DNA modifying enzymes (IHF and H-NS) and small regulatory RNAs [8],[11],[12],[13].

Curli mediate interactions between the bacteria and their environment. They play a critical role in the biofilm formation on biotic and abiotic surfaces, in cell-cell interactions and in pathogenesis [7],[14]. In *E. coli*, the ability to form biofilms is correlated with the strong increase of the initial adhesion in curli-overproducing bacteria [12]. In some *E. coli* strains, curli and cellulose are the major constituents of the biofilm extracellular matrix and so contribute to the bacterial resistance to environmental stresses and antimicrobials agents [15],[16].

Being the most biocide used in periodontology, the effectiveness of CHX-Dg has been particularly studied on supragingival bacteria. Nevertheless, although it is also used as disinfectant or antiseptic, few data are yet available in the literature on the impact of CHX-Dg on the biofilm formation and the bacterial physiology in *Enterobacteriaceae*. Consequently, we focused here on the effect of CHX-Dg treatment on the curli production and on the biofilm formation in *E. coli*. Moreover, in order to explain some observations, proteomic investigations and experiments with a Δ*Cpxr* mutant were performed.

## 2. Materials and Method

### 2.1. Bacterial culture

Four bacterial strains of *E. coli* were used in the present study: the K-12 MG1655 strain, a *cpxR-*null mutant (NR754-MC4100 ara+ Δ*cpxR:kan*) and the wild-type control NR754-MC4100 ara+. The *cpxR-*null mutant and the control strain (kindly provided by the Silhavy Lab in Princeton University) were used to evaluate the influence of the Cpx pathway on the curli expression. In the Δ*cpxR:kan* mutant, the *cpxR* ORF was replaced with a kanamycin-resistance cassette from plasmid pKD4 and the Met codon and the final six amino acid codons are preserved. The *cpxR*-null mutant was precultured in LB medium supplemented with 25 µg ml^−1^ kanamycin, to control the mutation maintenance. Curli being expressed in early stationary phase [9], all experiments were performed with overnight cultures carried out in 100 ml LB medium at 30 °C under agitation (120 r min^−1^, New Brunswick Scientific, USA). Moreover, the temperature being one of the main environmental conditions recognized to regulate the curli synthesis [9],[11], a temperature shock from 30 °C to 37 °C was carried out to investigate the impact of the temperature on the curli expression.

### 2.2. Determination of minimum inhibitory and bactericidal concentrations

The MIC and MBC were determined according to the procedures recommended by the Clinical and Laboratory Standards Institute. From an initial cell concentration of 10^6^ CFU ml^−1^, the standard microdilution technique with Mueller-Hinton Broth (MHB) and the bacterial enumeration on Mueller-Hinton Agar (MHA) were used. The MIC was defined as the lowest CHX-Dg concentration that inhibited the visible growth after incubation for 24 h. The MBC was defined as the lowest concentration of antiseptic killing 99.9% of the inoculum, which corresponded in our study to a bacterial count of less than 10^3^ CFU ml^−1^ after 24 h of incubation.

### 2.3. Influence of the temperature and CHX-Dg on curli production

Three culture conditions were performed with the 3 strains tested. After overnight incubation at 30 °C in 100 ml of LB medium under agitation, 10 µg ml^−1^ CHX-Dg were added in one of the three overnight cultures and all cultures were kept at 30 °C. After 2 h, one of the two cultures without antiseptic was used as control (C0) and was maintained at 30 °C. The second culture (C1) was transferred to 37 °C to highlight the adaptive response of the bacteria to the temperature shift. The culture with CHX-Dg (C2) was incubated as C1. The production of curli in planktonic cells was estimated by CR staining for 5 h 30 min after the addition of CHX-Dg, i.e. the minimal duration to observe a curli down-regulation at 37 °C (data not shown).

The CR-indicator plate provides a simple and useful method to assess curli production. The colonies of curliated bacteria strains are red when grown on YESCA agar plate supplemented with CR [5]. This assay was performed at 30 °C and 37 °C for 72 h with the MG1655 strain. However, this methodology was not applied for the C2 condition, CHX-Dg inhibiting the bacterial growth on the Petri dish. Therefore, a bacterial staining assay from liquid medium was undertaken. At 5 h 30 min, cultures (C0, C1 and C2) were centrifuged at 3500 × g for 10 min. The supernatant was removed. A volume of 5 ml of CR solution (0.5 g L^−1^ of CR in potassium phosphate buffer) for 0.1 g wet weight of bacteria were added and mixed. After a 5-min delay, cells (0.3 g) were centrifuged at 3500 × g for 10 min. The pellets were solubilized in 10 ml potassium phosphate buffer (50 mM, pH 7.2) for 0.3 g wet weight of bacteria. The amount of CR fixed on micro-organisms was determined by spectrophotometry at 490 nm, i.e., the maximum absorption wavelength of Congo red (Supplementary data, Figure S1a). The differences between the OD values obtained from stained and unstained bacterial solutions were integrated in a calibration curve, previously established with different CR concentrations (Supplementary data, Figure S1b). The amount of fixed dye was expressed as mg g^−1^ of bacteria. Experiments were performed in triplicate.

### 2.4. Crystal violet staining

The test was adapted from a method previously described [17]. One of 100 mL overnight culture, performed at 30 °C in LB medium, were deposited in each well of 24-well plates. The plates were incubated in the C0, C1 and C2 conditions under agitation. Adhesion assays were performed with different antiseptic concentrations, i.e., 1, 2, 5 and 10 µg ml^−1^. After 5 h 30 min, unattached cells were removed, diluted and spread on LB agar dishes for bacterial counts. Wells were rinsed thoroughly and slowly with water. Attached cells were subsequently stained by incubation with 1.5 ml 0.5% (w/v) Crystal Violet (CV) for 20 min. The CV was removed and the wells were carefully rinsed 3 times with 2 ml water. The cell-attached CV was then solubilized by adding 1.5 ml of absolute ethanol. The OD of the solution was measured at 570 nm. Experiments were performed at least in triplicate.

### 2.5. Outer membrane protein extraction

Crude outer membrane (OM) extracts of the MG1655 strain were prepared as previously described [18]. Bacteria were harvested by centrifugation for 30 min at 2600 × g, and washed with 20% (w/v) sucrose. Cells (ca. 1.5 g, wet weight) were suspended in a digestion solution consisting of 28 ml of 2 M sucrose, 10 ml of 0.1 M Tris-HCl (pH 7.8 at 25 °C), 0.8 ml of 1% EDTA and 1.8 ml of 0.5% (w/v) lysozyme. After 30 min of incubation, RNase and DNase (each at 3 µg ml^−1^, Sigma) were added. The mixture was incubated again for 1.5 h at 30 °C. Spheroplasts were eliminated by centrifugation (10,000 × g for 15 min) and the supernatant was collected. OMs were then pelleted by centrifugation at 80,000 × g for 40 min at 4 °C and resuspended in MilliQ water. The amount of proteins in the sample was evaluated using a Bradford protein assay. For each condition (i.e., C0, C1 and C2) subsequently submitted to the proteomic investigation, the extraction was performed in triplicate.

### 2.6. Trypsin digestion and nano LC-MS/MS

Digestion and nanoLC-MS/MS analyses were performed according to the procedures previously described [19]. Prior to the digestion, 25 µg of membrane extract were loaded on SDS-PAGE gel constituted of 6% polyacrylamide (width 16 cm, length 20 cm, thickness 0.75 mm). After migration for 1 h at 20 mA, proteins were stained with Coomassie blue. Protein bands were excised and incubated in a reductive solution of 5 mM dithiothreitol and alkylated in a 25 mM iodoacetamide solution. Bands were then washed several times with water and ammonium carbonate, dehydrated with acetonitrile and dried. Digestion was performed overnight with 1 µg of trypsin (Promega) per band. The gel fragments were subsequently incubated once for 15 min in 1% (v/v) trifluoroacetic acid and once in 100% ACN to allow extraction of peptides from the gel pieces. Supernatants were combined and dried.

For mass spectrometry analysis, protein digests were dissolved in 0.1% formic acid and 65 ng were injected in a linear ion trap-Orbitrap mass spectrometer (LTQ Orbitrap Elite, Thermo Scientific) equipped with a nano-ESI source coupled to a nanoliquid chromatography (Easy-nLC II, Thermo Scientific). Peptides were separated with a linear gradient of 15% to 55% of B (mobile phase A: H_2_O/0.1% FA and B: ACN/0.1% FA) over 120 min on a reversed phase C18 column (NikkyoTechnos, Japan) using a linear gradient. The mass spectrometer was operated in data dependent mode to automatically switch between Orbitrap-MS (from m/z 300 to 2000 with a resolution of 30,000) and LTQ-MS/MS acquisition.

### 2.7. Identification and quantification of proteins

Mass spectrometry data (raw data files) obtained from C0, C1 and C2 samples were processed using both Proteome Discoverer 1.3 (Thermo Scientific) and Progenesis LC-MS-MS (Nonlinear Dynamics) software packages.

For protein identification with Proteome Discoverer, peak lists were searched using the MASCOT search engine (Matrix Science) against the *E. coli* database. Searches were performed with the following parameters: 1 missed cleavage sites and variable modifications (carbamidomethylation of Cys and oxidation of Met). The parent ion and daughter ion tolerances were 10 ppm and 0.5 Da, respectively. Only peptides exhibiting significant Mascot individual ion score were retained. Due to the high sensibility of the label free approach, only identified protein exhibiting a sequence coverage upper to the third of the total sequence were considered.

Protein quantification with Progenesis LC-MS-MS software (4.0.4356.49980 version) was achieved according to Obry et al. [19]. Briefly, after alignment from one sample as reference and after normalization, PCA (Principal Component Analysis) was performed, in a first time, without statistic filters to interpret the global variations of protein quantity among the experimental conditions. In a second time, analysis of variance (ANOVA) with statistic filters, were performed to select the significant variation of protein expressions. The peptide signals exhibiting *P* value < 0.05 were conserved and the corresponding MS/MS spectra were exported for peptide identification with Mascot (Matrix Science v2.1.3) against the SwissProt database restricted to *E. coli*. The total cumulative abundance of the protein was calculated by summing the abundances of identified peptides. Proteins with a *q* value < 0.05, a power > 0.8 and quantified with at least 2 peptides were selected. In addition to the previous statistical parameters, a 1.8-fold ratio for significant spot alteration has been arbitrarily chosen. The label free experiments were performed in triplicate for each culture conditions.

### 2.8. STRING analysis

Protein-protein interaction map of proteins differentially expressed was generated with STRING software (http://string-db.org). All STRING data on predicted protein interactions were verified.

## 3. Results

### 3.1. Effect of CHX-Dg on the bacterial growth

The 3 strains exhibited close MIC and MBC values (see Table 1). The MIC values were lower than 1 µg ml^−1^ and appeared lower at 30 °C than at 37 °C. Conversely, the MBC values were greater at 30 °C than at 37 °C. Killing curves performed at 30 °C with CHX-Dg concentrations ranging from 1 to 20 µg ml^−1^ pointed out no bactericidal effect of the antiseptic at concentrations of 1 and 5 µg ml^−1^ (Supplementary data, Figure S2). Although the CMB was reached, a bacteriostatic effect was observed at 10 µg ml^−1^ CHX-Dg, probably because of the high initial cell concentration (2–3 × 10^9^ CFU ml^−1^). Consequently, the concentration of 10 µg ml^−1^ was retained for further experiments. Higher antiseptic concentrations (15 and 20 µg ml^−1^) induced a high killing effect.

**Figure 1.**
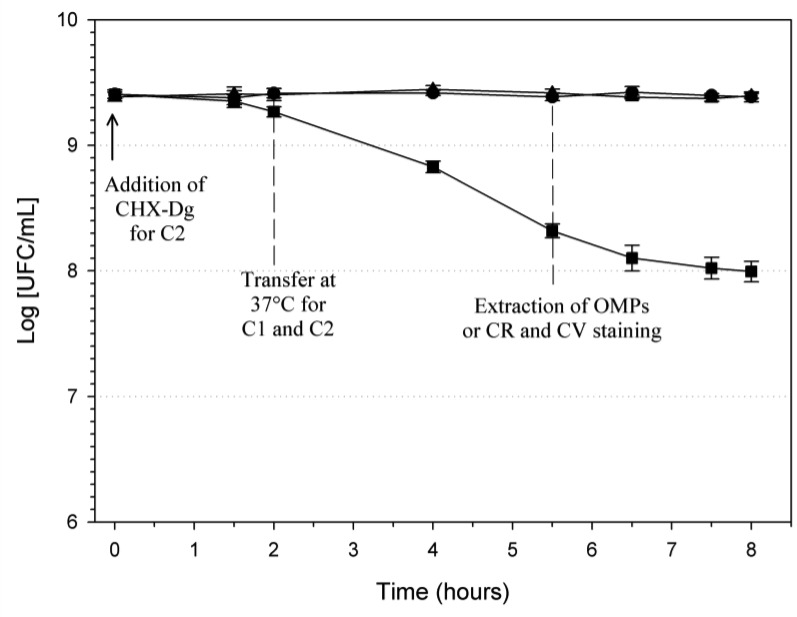
Influence of the temperature and 10 µg ml^−1^ CHX-Dg on the growth of *E. coli* MG1655 strain in LB broth. Incubation conditions: ▴, C0 (control at 30 °C without CHX-Dg); •, C1 (without CHX-Dg, at 30 °C for 2 hours and transfer to 37 °C); ▪, C2 (with CHX-Dg, at 30 °C for 2 hours and transfer to 37 °C). Bars: SE (n = 3).

The temperature shift from 30 °C to 37 °C had no notable effect on the bacterial growth kinetic (Figure 1). In presence of CHX-Dg, the antiseptic exhibited slight activity during the first 2 hours at 30 °C. However, the transfer to 37 °C enhanced the antimicrobial effect.

**Table 1.** Minimum inhibitory concentration (MIC) and minimum bactericidal concentration (MBC) values in µg/mL of Chlorhexidine-Dg, at 30 °C and 37 °C.

	30 °C		37 °C	

*E. coli* strains	MIC	MBC	MIC	MBC
K-12 MG1655	0.5	4	0.7	3
NR754-MC4100 ara+	0.6	4	0.8	3
NR754-MC4100 ara+ Δ*cpxR:kan*	0.4	4	0.7	3

### 3.2. Effect of the growth temperature and CHX-Dg on the curli production

Before investigations in liquid medium, the curli production in the MG1655 strain was controlled on CR-Yesca plates (Supplementary data, Figure S3). As expected, colonies were red stained at 30 °C, demonstrating curli production, whereas a whitish morphotype was observed at 37 °C, indicating a lack of curli production.

**Figure 2.**
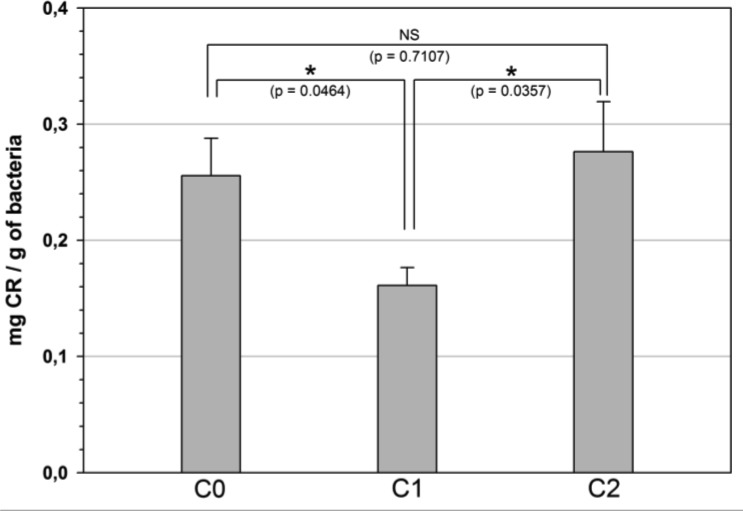
Influence of the temperature and 10 µg ml^−1^ CHX-Dg on the curli production in *E. coli* MG1655 strain. Quantity of Congo Red (CR) fixed on bacteria in the 3 culture conditions: C0 (control at 30 °C without CHX-Dg); C1 (without CHX-Dg, at 30 °C for 2 hours and transfer to 37 °C) and C2 (with CHX-Dg, at 30 °C for 2 hours and transfer to 37 °C). Bars: SE (n = 3). Stars indicated level of significance by *t*-test *P*-value (* *P* ≤ 0.05, ** *P* ≤ 0.01 and *** *P* ≤ 0.001) and NS: Not Significant.

**Figure 3.**
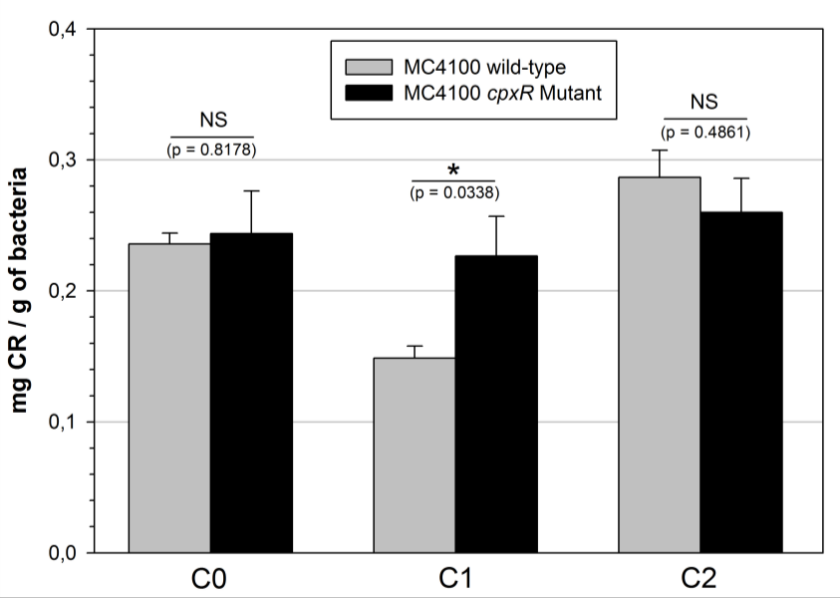
Influence of the *cpxR* mutation on the curli production in *E. coli* MC4100 strain. Quantity of Congo Red (CR) fixed on the bacteria incubated 5 h 30 min in the 3 culture conditions (C0: control at 30 °C without CHX-Dg; C1: without CHX-Dg at 30 °C for 2 hours and transfer to 37 °C; C2: with 10 µg ml^−1^ CHX-Dg, at 30 °C for 2 hours and transfer to 37 °C). Bars: SE (n = 4). Stars indicated level of significance by *t*-test *P*-value (* *P* ≤ 0.05, ** *P* ≤ 0.01 and *** *P* ≤ 0.001) and NS: Not Significant.

As for the bacterial growth assay, a sudden shift of temperature was performed after 2 h of incubation in order to observe the adaptive response of the bacteria after transfer to 37 °C. In liquid medium, the CR staining experiments (Figure 2) revealed a significant reduction of the curli production consecutively to the temperature up-shift. However, the addition of 10 µg ml^−1^ CHX-Dg abolished this inhibitory temperature effect. Indeed, the curli production returned similar to that observed at 30 °C. This phenomenon was also observed with the MC4100 wild-type control strain (Figure 3).

### 3.3. Effect of the growth temperature and CHX-Dg on the bacterial adhesion

**Figure 4.**
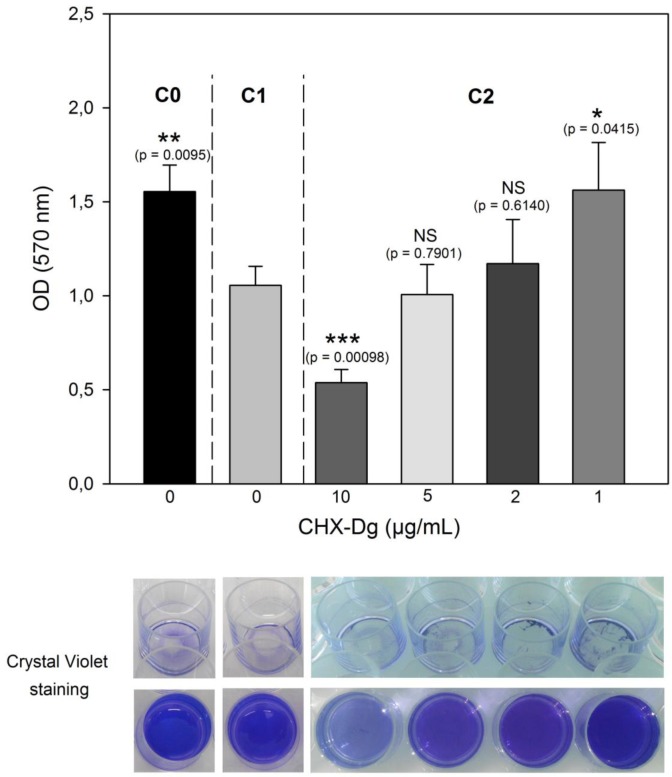
Influence of the temperature and the concentration of CHX-Dg on bacterial adhesion in *E. coli* MG1655 strain. Absorbance values at 570 nm from surface-attached bacteria stained with Crystal Violet (CV) and recovered in ethanol after 5 h 30 min: C0 (control at 30 °C without CHX-Dg), C1 (without CHX-Dg, at 30 °C for 2 hours and transfer to 37 °C) and C2 (with 1–10 µg ml^−1^ CHX-Dg, at 30 °C for 2 hours and transfer to 37 °C). Bars: SE (n = 5). Stars indicated level of significance by *t*-test *P*-value (* *P* ≤ 0.05, ** *P* ≤ 0.01 and *** *P* ≤ 0.001) and NS: Not Significant. Views of the wells containing CV stained adherent bacteria before and after recovery with ethanol.

A decrease of the bacterial adhesion was observed at 37 °C compared to 30 °C (Figure 4). Nevertheless, the presence of CHX-Dg at concentrations below 2 µg ml^−1^ (<MBC value) abolished this temperature effect. Thus, the adhesion ability of bacteria in presence of 1 µg CHX-Dg ml^−1^ was similar to that observed at 30 °C without CHX-Dg. For higher concentrations (>MBC value), the effect of the antiseptic did not allow to visualize this effect. The planktonic population was approximatively 2.07 × 10^9^ UFC ml^−1^ for C0 and C1 and 2.22 × 10^8^ UFC ml^−1^ for C2 in presence of 10 µg ml^−1^ CHX-Dg.

### 3.4. Effect of the growth temperature and CHX-Dg on the periplasmic proteins (PPs) and OMPs expression

As expected, protein extracts were enriched in OMPs. The sequence coverage scores were 2 times higher for OMPs (x = 1051 ± 130) compared to the other proteins identified with a lower pertinence (see details in Supplementary data, Table S1).

**Figure 5.**
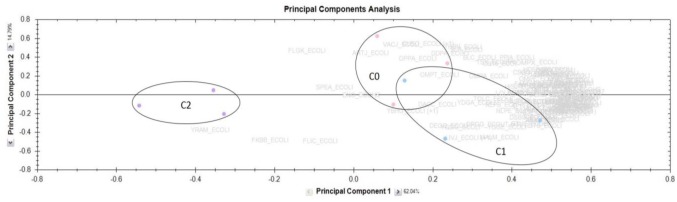
Principal Component Analysis performed without statistic filters from the 3 experimental conditions on all the identified OMPs and periplasmic proteins. C0 (control at 30 °C without CHX-Dg); C1 (without CHX-Dg, at 30 °C for 2 hours and transfer to 37 °C); C2 (with 10 µg ml^−1^ CHX-Dg, at 30 °C for 2 hours and transfer to 37 °C). Bars: SE (n = 3). Each colored circle represents one independent replicate. The names in grey represent the identified proteins.

The PCA performed from intensities of identified PPs and OMPs (Figure 5) and the quantitative proteomic analysis of C0 versus C1 (Supplementary data, Table S2), demonstrated that the temperature shift had no significant effect on the bacterial proteome. Indeed, the PCA discriminated only one component which explained 62.04% of the variation. This component could be attributed to the presence of CHX-Dg.

PPs and OMPs abundances were then compared according to the experimental conditions. Twelve OMPs and 32 PPs were modified in C2 as compared to C0 and C1 conditions (Table 2). Surprisingly, only one up-regulated protein (i.e., FklB) was observed in the presence of CHX-Dg. The majority of proteins which were down-regulated in the presence of the disinfectant, were involved in the transport of molecules, the envelope integrity, the stress response or the protein folding.

**Table 2.** Differential protein expression between the C2 (37 °C with 10 µg ml^−1^ CHX-Dg)/C1 (37 °C without CHX-Dg) and C2/C0 (30 °C without CHX-Dg) conditions. Biological processes and locations are assigned according UniProt and BioCyc databases: OM (Outer Membrane) and P (Periplasmic).

Biological process	Protein (Gene) in *Escherichia coli* K12	Function	UniProt Accession	Location	coverage (%) from identification	Peptides used for quantitation	Confidence score	Max fold change	
								C2 vs C1	C2 vs C0
								(−)*	(−)*
Transport	Outer membrane protein F (ompF)	Transport of small molecules—Receptor bacterophage T2	P02931	OM	86	5	372	2.94	
	Outer membrane protein X (ompX)	Implicated in secretion of extracellular protein (YebF) and adhesion	P0A919	OM	68	5	480	4.80	5.12
	Outer membrane protein C (ompC)	Transport of small molecules	P06996	OM	87	11	834	2.71	2.68
	Outer membrane protein A (ompA)	Transport of small solutes—Receptor for T-even like phages	P0A911	OM	70	4	294	3.69	3.06
	Periplasmic oligopeptide-binding protein (oppA)	Transport of peptides	P23843	P	80	8	740		2.56
	Periplasmic dipeptide Transport protein (dppA)	Transport of dipeptides—Chemotaxis (subject to osmotic shock)	P23847	P	75	11	554	3.78	4.79
	Antigen 43 (flu)	Auto Transporter—Controls autoaggregation	P39180	OM	53	6	577	2.07	
	Outer membrane lipoprotein slyB (slyB)	Transport of lipids	P0A906	OM	39	6	667	21.01	16.12
	Outer membrane protein W (ompW)	Component of colicin S4 transport system (receptor)	P0A915	OM	58	5	501	2.87	4.88
	Putative osmoprotectant uptake system substrate-binding protein OsmF (osmF)	Transport (involved in uptake of osmoprotectant molecules)	P33362	P	76	9	576	2.75	
	Putative ABC Transporter periplasmic-binding protein YdcS (ydcs)	Binding protein of a predicted spermidine/putrescine ABC Transporter	P76108	P	76	7	416	3.23	2.21
	Thiosulfate-binding protein (cysP)	Transmembrane transport of sulfate/thiosulfate (import)	P16700	P	53	7	391	3.41	3.15
	ABC Transporter periplasmic-binding protein (ytfQ)	Binding component of a galactose ABC transporter	P39325	P	38	6	386	2.37	
	Molybdate-binding periplasmic protein (modA)	Binding component of the molybdate ABC transporter	P37329	P	48	4	254		2.60
	Probable phospholipid-binding protein MlaC (mlaC)	Binding protein of the phospholipid ABC transporter (actively prevents phospholipid accumulation at the cell surface)	P0ADV7	P	63	4	279	3.67	
	Glutamine-binding periplasmic protein (glnH)	Transport of glutamine (system GlnHPQ)	P0AEQ5	P	69	7	354	3.20	2.17
	Maltoporin (lamB)	Transport of maltose and maltodextrins—Receptor several bacteriophages	P02943	OM	47	6	428	3.45	
	Cystine-binding periplasmic protein (fliY)	Transport of cyst(e)ine	P0AEN0	P	83	6	493	2.84	1.98
	Outer-membrane lipoprotein carrier protein (lolA)	Transport of proteins	P61316	OM	50	4	253	2.79	
	Arginine-binding periplasmic protein 1 (artI)	Transport of arginine	P30859	P	65	5	228	2.98	
	D-ribose-binding periplasmic protein (rbsB)	Transport of carbohydrate—Serve as primary receptor for chemotaxis	P02925	P	70	9	716	2.27	
	DcrB protein (dcrB)	Required for phage C1 adsorption	P0AEE1	P	66	4	181	4.18	
	Vitamin B12 transport periplasmic protein (btuE)	Part of btuCED operon. the vitamin B12 transport system	P06610	P	42	2	183	2.05

Stress response	Osmotically-inducible lipoprotein E (osmE)	Global regulatory functions	P0ADB2	OM	67	5	370	9.57	10.35
	Periplasmic serine endoprotease (degP)	Involved in proteolysis at elevated temperatures and protein folding (biogenesis of partially folded outer-membrane protein)	P0C0V1	P	64	16	1257	3.57	
	Osmotically-inducible protein Y (osmY)	Increases sensitivity to hyperosmotic stress—Induced upon entry into stationary phase	P0AFH9	P	54	8	808		2.36
	YgiW protein (ygiW)	Involved in the cellular response to hydrogen peroxide and cadmium stress	P0ADU6	P	42	2	86	10.16	8.70
	Acid stress chaperone HdeA (hdeA)	Required for optimal acid stress protection. Exhibits a chaperone-like activity only at low pH by suppressing non-specifically the aggregation of denaturated periplasmic proteins	P0AET0	P	55	2	151	33.23	30.4
	Thiol peroxidase (tpx)	Antioxidant activity	P0A864	P	79	4	223	2.40

Envelope integrity	Peptidoglycan-associated lipoprotein (pal)	member of the Tol-Pal system required for the maintenance of outer membrane stability	P0A913	OM	70	3	192		3.74
	Outer membrane protein slp (slp)	Stabilizes the outer membrane during carbon starvation and stationary phase	P37194	OM	55	2	127	2.37	2.15
	Probable L,D-transpeptidase YbiS (ybiS)	Responsible, at least in part, for anchoring of the major outer membrane lipoprotein (Lpp) to the peptidoglycan via a meso-diaminopimelyl-L-Lys- bond on the terminal residue of Lpp	P0AAY0	P	41	2	90	8.95	
	Uncharacterized protein ybgF (ybgF)	Involved in cell Envelope integrity—Role in the import of group A colicins and DNA of filamentous bacteria	P45955	P	70	5	257	3.88

Protein folding	Spheroplast protein Y (spy)	Chaperone that prevents protein aggregation and assists protein refolding	P77754	P	21	2	52	4.17	3.24
	FKBP-type peptidyl-prolyl cis-trans isomerase FkpA (fkpA)	PPIase that accelerates the folding of proteins	P45523	P	54	8	595	2.43	
	FKBP-type 22 kDa peptidyl-prolyl cis-trans isomerase FklB (fklB)	PPIase that accelerates the folding of proteins	P0A9L3	P	52	3	166		0.50
	Periplasmic peptidyl-prolyl isomerase SurA (surA)	PPIase involved in the correct folding and assembly of outer membrane proteins—May act in both early periplasmic and late outer membrane-associated steps of protein maturation	P0ABZ8	P	45	5	341	2.41	1.86
	Skp protein (skp)	Chaperone that interacts specifically with outer membrane proteins, thus maintaining the solubility of early folding intermediates during passage through the periplasm	P0AEU9	P	43	5	358	7.51	5.08
	Thiol:disulfide interchange protein DsbA (dsbA)	Required for disulfide bond formation in some periplasmic proteins such as PhoA or OmpA. Acts by transferring its disulfide bond to other proteins	P0AEG5	P	47	4	247	3.74	2.59

Metabolic process	L-asparaginase 2 (ansB)	Asparaginase activity	P00805	P	63	2	137		3.90
	Glucose-1-phosphatase (agp)	Glucose metabolic process—dephosphorylation	P19926	P	73	7	489		2.37

Signal	Autoinducer 2-binding protein LsrB (lsrB)	Part of the ABC Transporter complex LsrABCD involved in autoinducer 2 (AI-2) import (quorum sensing)	P76142	P	48	4	203	3.09	2.29

unknown	protein ytfJ (ytfj)	Uncharacterized: hypothesized to be involved in temperature stress	P39187	P	36	3	122	2.76	2.14
	protein yncE (yncE)	Uncharacterized: hypothesized to be involved in iron acquisition	P76116	P	34	3	181	3.09

* under-expression.

The STRING analysis revealed known and/or potential interactions between the differentially expressed proteins (Supplementary date, Figure S4). This analysis discriminated three groups. The main group clustered proteins which exhibit strong interactions, i.e., OmpA, OmpC, OmpF OmpX, OmpW, Skp, Deg P, DsbA, FkpA, LamB and SurA. These proteins are involved in the transport, folding, export and biogenesis of many PPs and OMPs. The second group concerned adaptation proteins, i.e., OsmY, OsmE, Slp, DcrB and HdeA. The last cluster grouped GlnH, DppA and RbsB, i.e., proteins involved in molecules transport (amino acids, peptides and carbohydrates, respectively).

### 3.5. Behaviour of the ΔcpxR mutant

Among the down-regulated proteins identified in the proteomic study, some of them (DegP, FkpA also named PpiA, DsbA and Spy) are up-regulated by the CpxA/R-TCS [13]. This Two-Component System is known to be involved in the negative regulation of curli expression [11],[12]. Therefore, the behaviour of a Δ*cpxR* mutant in the 3 experimental conditions, i.e., C0, C1 and C2, was investigated. As observed in Figure 3, whereas the MC4100 wild-type control strain displayed a drastic reduction of the curli production at 37 °C, the Δ*cpxR* mutant exhibited an identical curli production in the 3 culture conditions. In particular, non-abolishment of the curli production after the temperature up-shift was observed. The growth rates at 30 °C and 37 °C for the MG1655 and MC4100 strains (wild-type and mutant) and the curves of growth in the C0, C1 and C2 conditions were similar (Supplementary date, Figure S5a and S5b).

## 4. Discussion

### 4.1. Effect of CHX-Dg on the bacterial growth

The MIC levels measured on the three strains were low (about 0.5 µg ml^−1^) as compared with values reported for clinical strains [20]. The higher MICs in the environmental strains are probably due to the establishment of adaptation mechanisms. Furthermore, the rise in temperature at 37 °C increased the antimicrobial effect of 10 µg ml^−1^ CHX-Dg while a bacteriostatic effect was observed at 30 °C for the same antiseptic concentration. A hypothesis to explain this observation might be a change in the membrane fluidity, in particular a modification of the ratio of unsaturated to saturated fatty acids [21]. Such changes in the lipid composition might indeed affect the penetration of CHX-Dg into the cell. Further investigations would be necessary to confirm this hypothesis.

### 4.2. Impact of CHX-Dg on the curli production and on the bacterial adhesion

In most laboratory *E. coli* strains, the curli expression was induced below 30 °C and the cellulose production was absent whatever the temperature conditions. In accordance with the bibliographic data [6],[10], the present study pointed out the inhibition of the curli synthesis at 37 °C. The outstanding result is here the maintenance of the curli production at 37 °C in the presence of CHX-Dg. A similar observation was reported in *E. coli* planktonic cells grown at 37 °C in the presence of ciprofloxacin, amikacin and colistin [22]. However, no action mechanism has been yet identified to explain this effect observed at low concentrations of antimicrobials. Their identification required obviously more investigations.

Curli are critical determinants of biofilm formation; they mediate initial surface attachment and/or contribute to biofilm integrity [15],[16],[23],[24]. Some previous studies showed that curli-producing *E. coli* strains form more biofilms on polyurethane or polystyrene surfaces than curli-deficient ones [12],[23]. In accordance with these data, a higher bacterial adhesion at 30 °C was observed in absence of CHX-Dg. No positive impact of CHX-Dg on the ability of bacteria to adhere at 37 °C was observed at concentrations above 2 µg ml^−1^, due to the antimicrobial activity of the antiseptic. However, the adhesion capacity of bacteria in presence of 1 µg ml^−1^ of antiseptic was similar to that observed at 30 °C without CHX-Dg. Such biofilm induction in response to a broad range of antibiotics at low-concentrations was described in many bacteria including *E. coli* [25]. An increase of the biofilm formation by sublethal concentrations of chlorhexidine has also been described in *Staphylococcus epidermidis* [26]. Today, the mechanisms enhancing the biofilm development in presence of antibiotics and antiseptics are not well known but may result from a global stress response.

### 4.3. Impact of CHX-Dg on the PPs and OMPs expression

In the absence of antiseptic, the temperature shift didn't alter the bacterial growth and had none significant effect on the protein expression (see Table S2). This last observation is quite surprising since it is well known that the temperature is a cue to the regulation of the bacterial gene expression. Thus, White-Ziegler et al. [27], using DNA microarrays, identified 297 genes whose expression was increased at 23 °C compared to 37 °C in *E. coli* K-12. Among these genes, 122 were known to be controlled by RpoS. Another category of genes highly expressed at 23 °C were genes associated with the biofilm development, among which *csgBA*. This discrepancy is probably due to major differences in the experimental conditions, e.g., the growth phase (the early-stationary-phase in the present work and the mid-exponential phase for White-Ziegler et al.), the temperature shift value (7 °C here compared with 14 °C) and the fact that correlation between transcriptomics and proteomics approaches is often low [28].

Numerous studies devoted to the effectiveness of CHX-Dg are found in the literature but few works [29],[30] have focused on the impact of this antiseptic on the OMP profile, though the outer membrane is the cellular compartment in the immediate proximity to the extracellular environment and so the most sensible to environmental changes. After observations on the CHX-Dg impact on the curli production, proteomic investigations on the bacterial PPs and OMPs were performed. The PCA confirmed the low impact of the temperature shift on the protein expression, and pointed out significant changes in some periplasmic and outer membrane proteins amounts after the antiseptic treatment. None OMPs or PPs, constitutive of curli, was identified in the present investigation; probably due to the insoluble property of these appendages (maintaining of the amyloid-like structure). Nevertheless, three groups of affected proteins were discriminated: chaperones and porins (group 1), stress response proteins (group 2) and amino acid, peptide and carbohydrate transporters (group 3).

CHX-Dg altered the amount of several key periplasmic chaperones, e.g., SurA, DsbA, FkpA, Skp and DegP, which are essential for the folding, export and biogenesis of outer membrane proteins. These proteins were clustered in the group 1. The SurA pathway is described as the major pathway. The secondary pathway (DegP and Skp) serves in a back-up folding pathway. FkpA assists Skp in the assembly of some β-barrel proteins [31]. A previous label-free proteomic study in *E. coli* showed that 8 β-barrel proteins were negatively affected in a *surA* knockout mutant i.e., FadL, FecA, LptD, FhuA, OmpX, and OmpA, OmpF and LamB [32]. In agreement with these observations, we found here a down-regulation of OmpX, OmpA, Omp F and LamB in the presence of CHX-Dg, in addition to that of SurA (Table 1). Three other periplasmic chaperones were also down-regulated in the presence of CHX-Dg: LolA, involved in the transport of OM lipoproteins across the periplasm, DsbA involved in disulfide bond formation of many proteins (e.g., OmpA) and Spy recently highlighted as a periplasmic chaperone [31]. The results showed that CHX-Dg led to the under-expression of many chaperones mediating the proper localization of membrane and secretory proteins, an essential function for the bacteria survival [33]. Among these proteins, DegP, FkpA (PpiA), DsbA and Spy are positively controlled by the Cpx pathway that contributes with σ^E^ to the envelope integrity maintenance [13],[34]. These data show that CHX-Dg and the Cpx-TCS have antagonist effects on the production of these proteins.

The protein group 2 clusters polypeptides involved in the adaptation. Thus, the amount of HdeA, a key factor in *E. coli* acid resistance, drastically decreased in the presence of antiseptic. This protein is a stress-induced chaperone. HdeA promotes the resolubilization and the refolding of acid-denatured proteins and non-specifically suppresses the aggregation of proteins at low pH [31]. Recently, Surmann et al. showed that the production of this protein was positively controlled by the Cpx-TCS [35]. This down-production of HdeA might suggest a lower acid resistance for bacteria in contact with CHX-Dg. In *E. coli*, differences in the sensibility to the acidic stress have been reported between curliated and not curliated strains. Nevertheless, this discrepancy was not directly correlated to the presence of curli [36]. Slp was also down-regulated in the presence of CHX-Dg. This lipoprotein is known to stabilize the OM in response to carbon starvation during the stationary phase [37]. Associated to HdeA (and YhiF), Slp protects the cell against pH-dependent toxic effects of metabolites [38]. The amount of two osmotic stress response proteins, i.e., OsmE and OsmY, also decreased in the presence of CHX-Dg. OsmE belongs to the family of the osmotically inducible and growth phase-dependent proteins. OsmY is implied in hyperosmotic resistance. The expression of these proteins has been reported as under the control of RpoS [39]. However, Conter et al. observed an osmotically inducible expression of *osmE* in the absence of functional *rpoS* gene [40].

Many amino acid, peptide and carbohydrate transporters are also impacted by CHX-Dg. These proteins are clustered in the group 3. Thus, DppA, a dipeptide transporter required for peptide chemotaxis, and GlnH, a glutamine transporter subunit of system GlnHPQ, were down-regulated. These 2 transporters, as many other transporters here identified, e.g., FliY, ArtI, and ModA (Table 1), are involved in protein homeostasis of the bacterial envelope [33]. The amount of SlyB was also strongly down-regulated at 37 °C in the presence of CHX-Dg, as compared to other growth conditions. SlyB is a small OM lipoprotein of 155 amino acids, well conserved in different Gram-negative bacteria. No clear function has been assigned to this protein so far. An accumulation of SlyB might result in a permeabilization of the OM, allowing non-specific siderophore uptake [41]. It has been also proposed that SlyB contributes to the integrity of the cell envelope in *Burkholderia multivorans* [42].

The under-expression of many chaperones and transporters in the presence of CHX-Dg might induce strong damage of membrane protein biogenesis and so an alteration of the cellular wall integrity. Such hypothesis accords well with observations by Cheung et al. [29] who pointed out morphological changes in *E. coli* in the presence of a bactericidal concentration of CHX-Dg. After exposure to the antiseptic, the cytoplasmic membrane was detached from the cell, forming bulges. Leakage of cellular content then occurred, conducting to the formation of ghost cells. Likewise, the under-expression of many OMPs in the presence of CHX-Dg might be the witness of a cell lysis. Recently, Murata et al. [43] proposed a model of sigma E-directed cell lysis, named PCD (Programmed Cell Death). Under stress conditions, the sigma E-directed response leads to a repair of damaged cells or a PCD cascade, depending on the extent of cellular damages. During the PCD cascade, cells stop the synthesis of OMPs, e.g., OmpA, OmpC and OmpW. In coherence with this PCD, the down-regulation of several stress-induced proteins was observed here.

Finally, it is interesting to note that FklB is the only protein that was accumulated at 37 °C in the presence of the antiseptic (Table 1). FklB is the newest member of the periplasmic PPIases in *E. coli* and contributes to about 1% of the total PPIase activity [44]. Few data are yet available on the regulation and function of this protein, however. Therefore, it is difficult here to speculate on the molecular mechanisms involved in its accumulation.

### 4.4. Impact of cpxR mutation on the curli production

An interesting data brought by the proteomic study is that the amount of some CpxA/R-TCS up-regulated proteins (e.g., DegP, DsbA and Spy) were altered in the presence of CHX-Dg. TCSs allow bacteria to rapidly respond to environmental conditions. They typically consist of a sensor histidine kinase and a response regulator. The sensing of a stimulus results, in a first time, in an autophosphorylation of the kinase and, in a second time, in the transfer of the phosphoryl group to the regulator. The phosphorylated regulator then mediates the cellular response by acting as a transcription factor of target genes [35]. The Cpx-TCS, comprised of the CpxA membrane kinase and the CpxR regulator, is involved in the cellular response to many environmental stimuli, e.g., perturbations of the cell envelope, salt, alkaline pH, attachment to abiotic surfaces [35]. The curli synthesis is down-regulated by the Cpx-pathway in response to high osmolarity, acid shock, heat shock, nutrient limitation, cell density and curlin accumulation [12]. In response of one or a combination of these factors, CpxR is phosphorylated by CpxA and repressed the transcription of the two *csg* operons [8],[13]. Due to the importance of this Cpx-pathway in the regulation of curli expression and its response to many stimuli, the impact of a *cpx* mutation on the temperature control of the curli production was investigated. CR staining experiments performed on the control MC4100 strain and the Δ*cpx* mutant pointed out that the mutation abolishes the temperature control of the curli production. This result demonstrates, for the first time, that the control of the curli expression by the temperature is Cpx-dependent. However, it was not possible to evaluate here the role of this TCS in the CHX-Dg effect at 37 °C. Its role cannot be turn down. The *csgDEFG* promoter is recognized as one of the most complexly regulated promoters in *E. coli* [45] and controlled by many pathways (e.g. RpoS, EnvZ-OmpR, RcsABCD). Further studies with some corresponding mutants might be interesting to provide additional information.

## 5. Conclusion

The present study demonstrates that the addition of a low concentration of CHX-Dg maintained the curli production at 37 °C and affected the cellular envelop composition of *E. coli* cells. The maintaining of the curli production in response to unhealthy conditions could be a survival solution used by the cell to improve the adhesion capacity to counteract the antimicrobial action. The high chlorhexidine concentrations commonly used in surface solutions (2–4%) limit the clinical implications of these data, which present a fundamental interest however. Nevertheless, it has been recently shown that medically relevant concentrations of chlorhexidine [0.1% and 1% (w/v)] increased the stiffness of *E. coli* biofilms [46].
