# Supplementary material for: Impact of chlorhexidine digluconate and temperature on curli production in Escherichia coli—consequence on its adhesion ability
Source: AIMS Microbiol. 2017 Dec 1;3(4):915–37. doi: 10.3934/microbiol.2017.4.915 (PMC6604964; doi:10.3934/microbiol.2017.4.915)
Supplement: Supplementary file 1 [file microbiol-03-04-915-s001.pdf]

AIMS Microbiology

AIMS Microbiology, 3(4): 915-937.
DOI: 10.3934/microbiol.2017.4.915
Received: 06 July 2017
Accepted: 27 November 2017
Published: 01 December 2017

http://www.aimspress.com/journal/microbiology

Research article

Impact of chlorhexidine digluconate and temperature on curli production in *Escherichia coli*—consequence on its adhesion ability

Laurent Coquet [1,*], Antoine Obry [2], Nabil Borghol [3,4], Julie Hardouin [1], Laurence Mora [4], Ali Othmane [3], and Thierry Jouenne [1]

[1] CNRS UMR6270, Normandie University, UNIROUEN, Plate-forme PISSARO, Mont-Saint-Aignan, France
[2] INSERM U905, Normandie University, UNIROUEN, Plate-forme PISSARO, France
[3] Biophysical Laboratory, Faculty of Medicine of Monastir, Monastir, Tunisia
[4] INSERM U1148, Laboratory for Vascular Transitional Sciences, Galileo Institute, Paris 13 University, Sorbonne Paris Cité, Villetaneuse, France

* **Correspondence:** Email: laurent.coquet@univ-rouen.fr; Tel: +33-2-35-14-60-12.

a

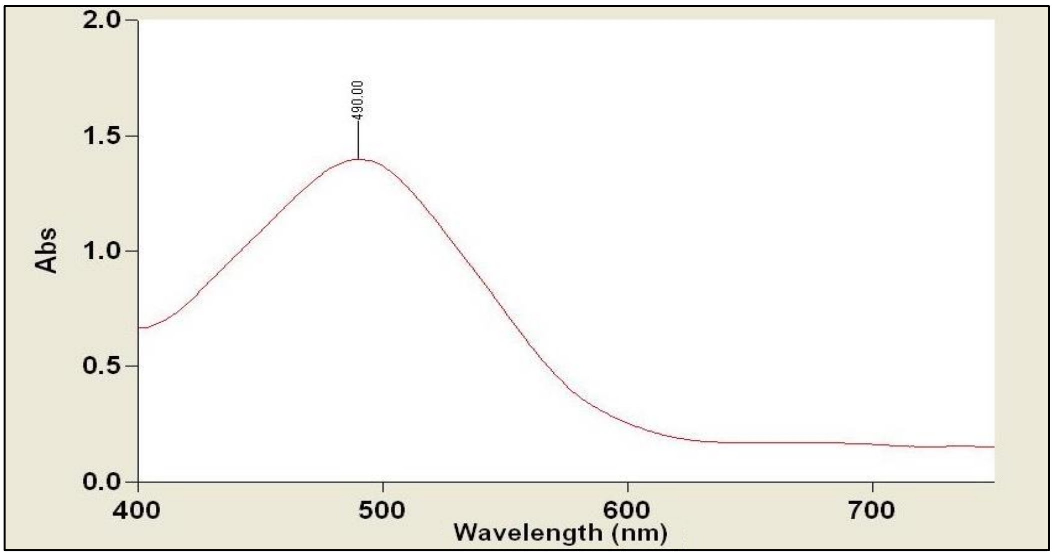

b

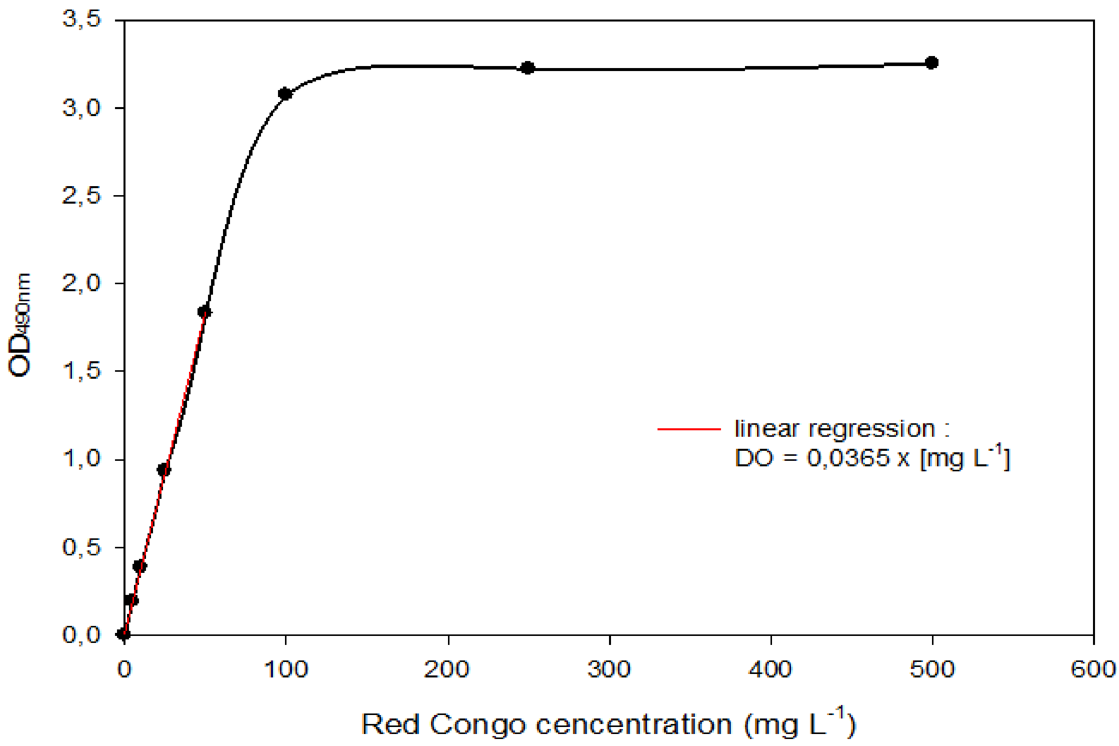

Figure S1. (a) Wavelength of maximum absorbance of Congo Red (scan from 400 to 750 nm with solution 500 mg CR l^{-1} diluted at 1/15). (b) Calibration curve of Congo Red at λ = 490 nm.

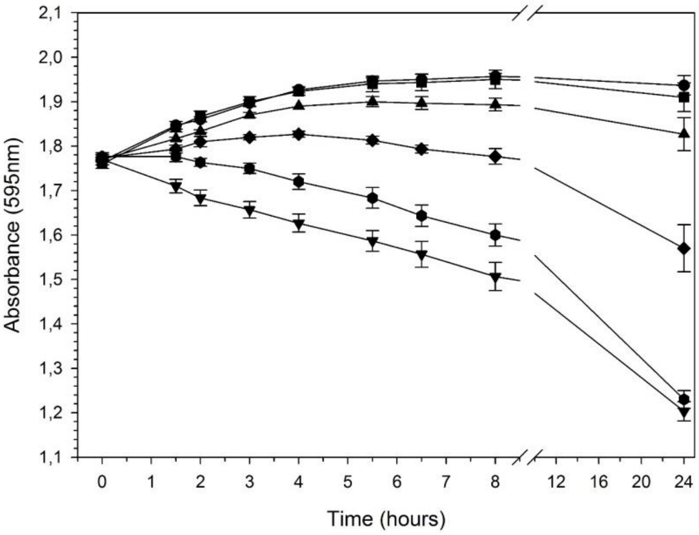

Figure S2. Kinetic of growth at 30 ℃ in LB with the *E. coli* MG1655 strain. Growth without (●, Control) and in presence of various concentration of CHX-Dg (■, 1 µg ml^{-1}; ▲, 5 µg ml^{-1}; ◆, 10 µg ml^{-1}; ⬢, 15 µg ml^{-1} and ▼, 20 µg ml^{-1}). Bars: SE (n = 3).

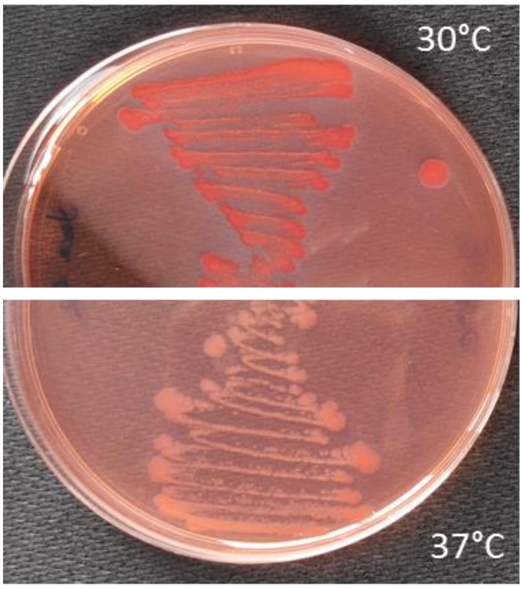

Figure S3. Phenotype of *Escherichia coli* K12-MG1655 on CR-Yesca plates after 72 h of incubation at 30 ℃ and 37 ℃.

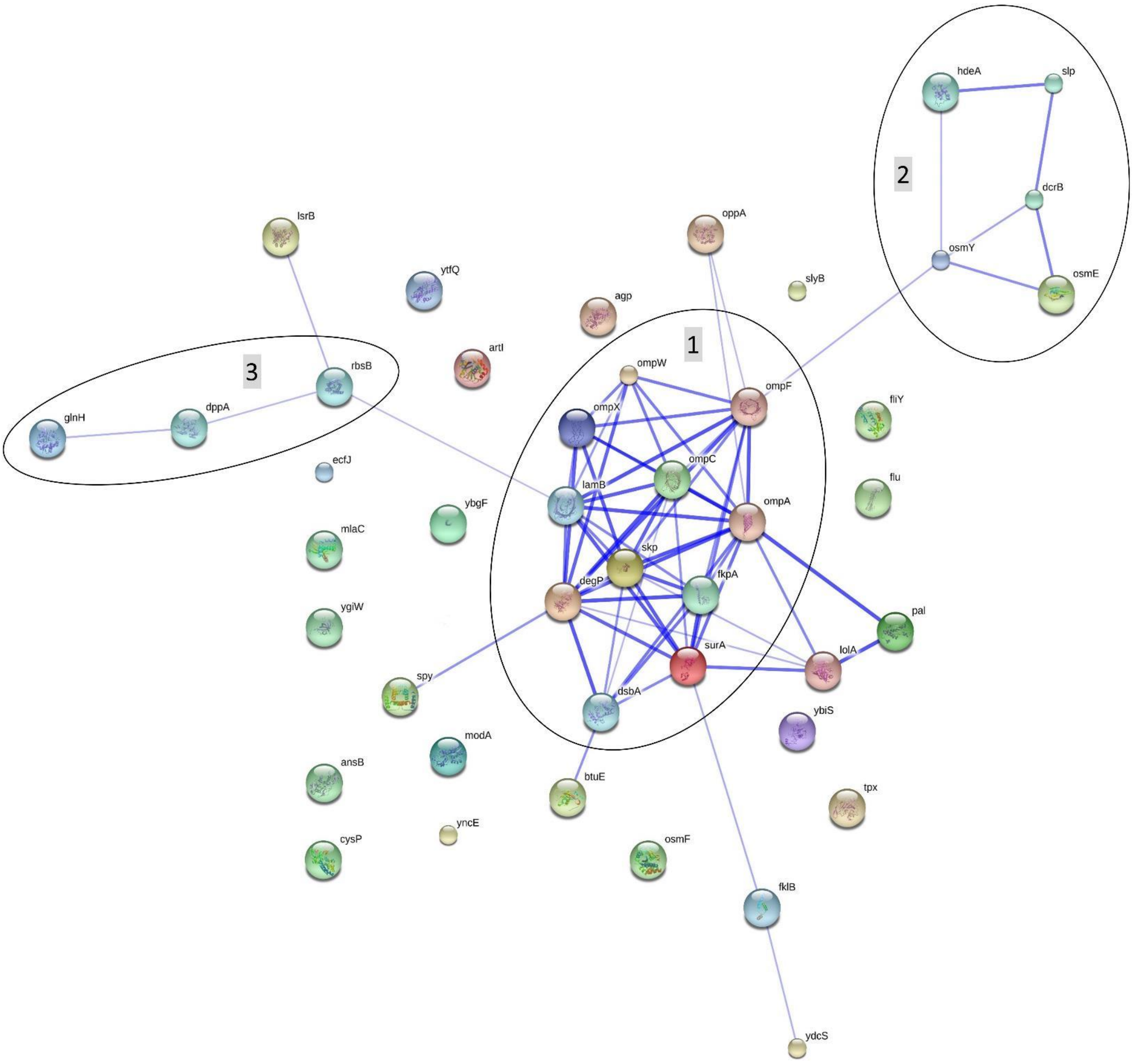

Figure S4. Protein-protein interaction map obtained with a medium confidence score (0.400) from proteins differentially expressed and identified in *E. coli* MG1655. The stronger associations are represented by thicker lines. Proteins implied (1) in transport, folding, export and biogenesis of outer membrane proteins, (2) in stress response and (3) in amino acid, peptide and carbohydrate transport.

a

b

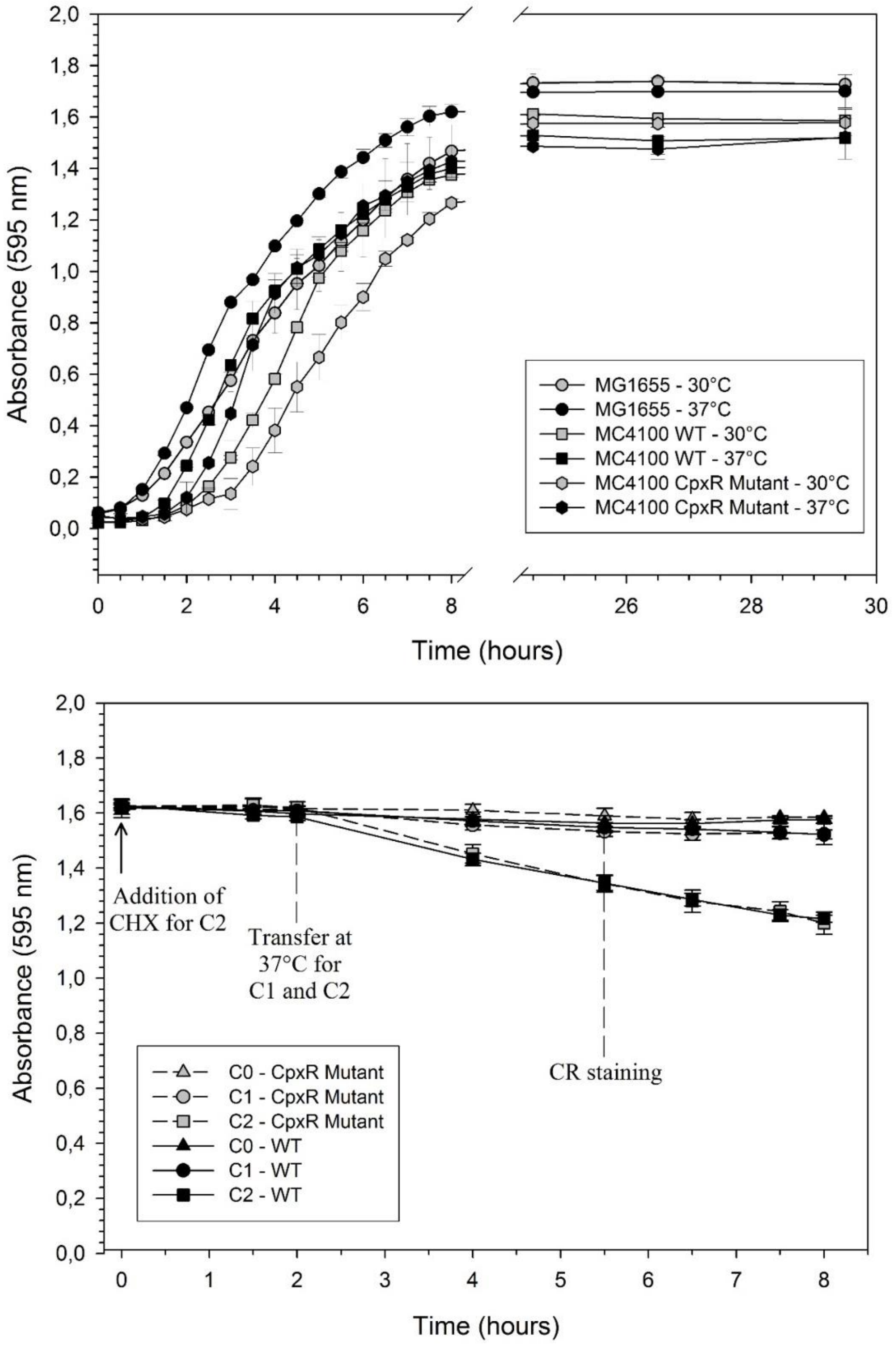

Figure S5. (a) Growth curves of the MG1655 and MC4100 (Wild Type and *cpxR*-null mutant) strains at 30 ℃ and 37 ℃ in LB broth. Bars: SE (n = 3). (b) Influence of the temperature and CHX-Dg on the growth of wild-type and *cpxR*-mutant MC4100 in LB broth. Incubation conditions: ▲, ▲, C0 (control at 30 ℃ without CHX-Dg); ●, ● , C1 (without CHX-Dg, at 30 ℃ for 2 hours and transfer to 37 ℃); ■, ■ , C2 (with CHX-Dg, at 30 ℃ for 2 hours and transfer to 37 ℃). Bars: SE (n = 3).

Table S1. Identified proteins in C0 (30 ℃), C1 (37 ℃ without CHX-Dg) and C2 (37 ℃ with CHX-Dg) by using the Proteome Discoverer software for the qualitative analysis. Cellular locations are assigned according UniProt and BioCyc databases: E (Extracellular), CS (Cell Surface), OM (Outer Membrane), P (Periplasmic), IM (Inner Membrane), C (Cytosolic and/or Cytoplasmic) and ND (Not Defined).

UniProt Accession	Proteins (*Escherichia coli*)	Cellular location	Σ Coverage	Σ# Peptides	Σ# PSMs	Score C0(1)	Coverage C0(1)	# Peptides C0(1)	# PSM C0(1)	Score C0(2)	Coverage C0(2)	# Peptides C0(2)	# PSM C0(2)	Score C0(3)	Coverage C0(3)	# Peptides C0(3)	# PSM C0(3)	Score C1(1)	Coverage C1(1)	# Peptides C1(1)	# PSM C1(1)	Score C1(2)	Coverage C1(2)	# Peptides C1(2)	# PSM C1(2)	Score C1(3)	Coverage C1(3)	# Peptides C1(3)	# PSM C1(3)	Score C2(2)	Coverage C2(2)	# Peptides C2(2)	# PSM C2(2)	Score C2(3)	Coverage C2(3)	# Peptides C2(3)	# PSM C2(3)	Score C2(4)	Coverage C2(4)	# Peptides C2(4)	# PSM C2(4)	# AAs	MW [kDa]	calc. pI	Σ# Detections in C0-C1-C2
P04949	Flagellin - [FLIC_ECOLI]	CS	92	31	1748	7874	91	29	211	4497	92	31	106	5176	89	26	143	6072	89	26	154	6724	89	26	181	6876	90	28	184	9583	90	28	264	8651	90	27	237	9706	90	28	268	498	51	5	3-3-3
P75937	Flagellar hook protein flgE - [FLGE_ECOLI]	CS,OM	38	9	68	461	38	9	11	250	22	5	5	263	21	6	6	391	27	6	8	400	30	7	7	320	30	7	7	277	24	6	8	351	24	6	7	382	27	7	9	402	42	5	3-3-3
P39180	Antigen 43 precursor - [AG43_ECOLI]	E, CS, OM, P	53	32	248	1189	43	26	30	1101	35	21	27	1006	33	20	23	1693	40	25	37	1286	37	24	31	950	33	19	22	971	34	20	24	1046	37	22	26	1267	37	22	28	##	107	6	3-3-3
A7ZQM2	Enolase - [ENO_ECO24]	E, CS, OM,IM,C	78	23	221	780	52	16	20	1069	61	18	28	869	47	14	22	1086	59	17	29	855	46	14	21	817	43	13	19	849	48	15	22	1219	59	19	32	1090	66	18	28	432	46	5	3-3-3
P10384	Long-chain fatty acid transport protein precursor - [FADL_ECOLI]	OM	36	9	65	317	34	8	8	319	25	7	8	364	31	7	9	299	24	6	7	287	24	6	7	280	24	6	7	201	11	4	4	272	24	6	7	336	24	7	8	446	49	5	3-3-3
P06129	Vitamin B12 transporter btuB precursor - [BTUB_ECOLI]	OM	36	15	65	615	36	15	18	89	8	3	4	232	14	6	7	388	22	9	12	348	14	6	10	139	15	7	7					52	5	3	3	180	8	3	4	614	68	5	3-3-2
P0A906	Outer membrane lipoprotein slyB precursor - [SLYB_ECO57]	OM	39	5	57	457	39	4	7	467	39	4	10	359	39	4	7	381	39	4	7	621	39	5	14	299	39	4	6	115	25	2	2					157	39	4	4	155	16	9	3-3-2
P0AC04	Outer membrane protein assembly factor BamC - [BAMD_ECO57]	OM	39	7	19	65	17	3	3													33	8	2	2					39	9	2	2	139	26	4	4	225	39	7	8	245	28	7	1-1-3
P77774	Outer membrane protein assembly factor BamB - [BAMB_ECOLI]	OM	40	9	45	362	32	7	8	229	21	4	5	165	12	3	3	260	20	5	6	201	12	3	4	159	12	3	3	173	12	3	3	226	20	5	5	294	35	8	8	392	42	5	3-3-3
P0A928	Nucleoside-specific channel-forming protein tsx precursor - [TSX_ECO57]	OM	40	7	24	262	28	5	7	160	25	4	4					113	22	4	4	78	8	2	2	101	8	2	2									141	24	5	5	294	34	5	2-3-1
P0A903	Outer membrane protein assembly factor BamC - [BAMC_ECOLI]	OM	43	8	41	284	32	6	8	167	13	3	4	201	26	5	5	184	22	5	5	251	17	4	6	159	26	5	5	90	9	2	2	132	15	3	3	126	15	3	3	344	37	6	3-3-3
P09169	Protease 7 precursor - [OMPT_ECOLI]	OM	46	13	81	425	36	10	12	284	30	8	9	263	27	7	8	298	38	11	11	307	27	8	9	173	20	5	5	222	21	6	6	280	27	8	8	423	44	11	13	317	36	6	3-3-3
A7ZKY3	Outer-membrane lipoprotein lolB precursor - [LOLB_ECO24]	OM	47	7	22	151	42	6	6					125	32	4	4	133	32	4	4	140	27	3	4													96	29	4	4	207	24	9	2-2-1
P02943	Maltoporin precursor - [LAMB_ECOLI]	OM	47	14	146	874	42	11	21	679	34	11	17	1008	40	10	18	943	40	10	20	812	40	10	17	690	37	9	14	343	21	6	8	410	36	10	12	759	43	11	19	446	50	5	3-3-3
A7ZJW0	Outer-membrane lipoprotein carrier protein precursor - [LOLA_ECO24]	OM	50	7	45	290	45	6	7	323	27	4	8	172	28	4	4	241	41	6	6	281	41	6	7	195	28	4	4	180	28	4	4	67	13	2	2	176	23	3	3	203	22	7	3-3-3
P37194	Outer membrane protein slp precursor - [SLP_ECOLI]	OM	55	6	31	197	39	4	6	78	25	3	3	74	30	4	4	159	30	4	5	72	30	4	4	44	20	3	3									169	45	5	6	188	21	7	3-3-1
P0A902	Outer membrane lipoprotein blc precursor - [BLC_ECO57]	OM	57	10	52	233	52	8	9	248	47	7	8	138	42	6	6	124	47	7	7	195	46	7	7	84	21	4	4	124	26	5	5	51	16	2	2	111	31	4	4	177	20	9	3-3-3
P0A915	Outer membrane protein W precursor - [OMPW_ECOLI]	OM	58	7	79	420	35	5	13	533	48	6	17	336	35	5	8	514	45	6	14	283	45	6	10	85	18	3	3	182	24	4	5	122	29	4	4	210	35	5	5	212	23	7	3-3-3
P69778	Major outer membrane lipoprotein precursor - [LPP_ECO57]	OM	58	4	58	200	58	4	7	299	49	3	8	201	58	4	6	226	49	3	6	225	49	3	6	223	49	3	6	197	49	3	6	199	49	3	5	270	58	4	8	78	8	9	3-3-3
P02930	Outer membrane protein tolC precursor - [TOLC_ECOLI]	OM	59	18	108	795	58	17	22	314	18	4	5	314	27	9	10	820	46	14	19	571	30	9	13	261	30	8	8	247	21	6	6	447	26	9	12	538	27	10	13	493	54	6	3-3-3
P0A919	Outer membrane protein X precursor - [OMPX_ECO57]	OM	68	10	431	1910	68	10	65	2162	68	10	60	1166	68	10	44	3356	68	9	97	2114	68	10	80	1374	68	9	51	452	63	7	13	248	44	6	6	548	49	7	15	171	19	7	3-3-3
P0A913	Peptidoglycan-associated lipoprotein precursor - [PAL_ECO57]	OM	70	7	52	365	70	7	9	222	61	6	6	249	61	6	7	252	61	6	7	221	40	4	5	190	32	3	4	88	21	3	3	94	21	3	3	235	51	6	8	173	19	7	3-3-3
P0A911	Outer membrane protein A precursor - [OMPA_ECO57]	OM	70	20	771	3642	70	20	133	2261	70	19	82	1623	61	18	59	3150	61	18	115	1828	61	19	68	1373	59	17	54	1555	60	18	57	2144	60	18	79	3192	61	17	124	346	37	6	3-3-3
P02931	Outer membrane protein F precursor - [OMPF_ECOLI]	OM	86	22	723	3290	77	21	122	3491	79	20	94	1770	63	17	56	3110	75	20	111	2466	75	20	83	1916	75	20	62	1875	75	18	69	1449	72	19	46	2442	75	19	80	362	39	5	3-3-3
P06996	Outer membrane protein C precursor - [OMPC_ECOLI]	OM	87	23	1306	6292	87	22	187	6193	87	23	170	3970	78	20	135	7503	87	22	226	4662	78	20	169	3109	78	21	105	2485	71	17	87	2609	71	20	89	4275	82	20	138	367	40	5	3-3-3
P76116	Uncharacterized protein yncE precursor - [YNCE_ECOLI]	P	33	6	24	177	10	2	2	150	24	3	3	257	15	3	4	210	16	4	4	228	18	4	4	159	18	4	4	143	13	3	3									353	39	9	3-3-1
P31133	Putrescine-binding periplasmic protein precursor - [POTF_ECOLI]	P	34	9	49	275	34	9	9	150	9	3	4	77	12	4	4	239	29	7	7	243	17	5	6	158	16	5	5	71	12	4	4	55	8	3	3	145	24	7	7	370	41	6	3-3-3
P75694	UPF0379 protein yahO precursor - [YAHO_ECOLI]	P	34	2	7	116	34	2	2	206	34	2	3									139	34	2	2																	91	10	6	2-1-0
P39187	Uncharacterized protein ytfJ precursor - [YTFJ_ECOLI]	P	36	5	38	245	32	4	6	120	20	3	4	110	32	4	5	101	15	2	3	150	32	4	4	128	32	4	4	109	20	3	5	80	20	3	3	77	20	3	4	184	20	8	3-3-3
P09394	Glycerophosphoryl diester phosphodiesterase precursor - [GLPQ_ECOLI]	P	37	8	32	164	25	5	6	217	27	5	7	49	5	2	2	137	18	4	5	125	13	3	4	49	5	2	2	51	11	2	2	48	5	2	2	82	7	2	2	358	41	6	3-3-3
P07024	Protein ushA precursor [Includes: UDP-sugar hydrolase - [USHA_ECOLI]	P	37	14	86	350	22	8	11	331	25	8	8	228	13	5	6	331	19	7	8	454	28	11	13	415	31	12	13	386	19	8	10	295	17	7	9	312	17	7	8	550	61	6	3-3-3
P0AGD2	Superoxide dismutase [Cu-Zn] precursor - [SODC_ECO57]	P	38	6	22	171	38	5	6	100	33	4	4	76	20	3	3	114	29	3	4	90	20	3	3	71	16	2	2													173	18	6	3-3-0

P39325	ABC transporter periplasmic-binding protein ytfQ precursor - [YTFQ_ECOLI]	P	38	10	67	370	33	8	11	293	31	7	8	168	28	6	6	266	31	7	8	262	28	6	7	237	25	7	7	230	25	7	8	219	23	6	6	228	23	6	6	318	34	7	3-3-3
A7ZKF2	Glucans biosynthesis protein G precursor - [OPGG_ECO24]	P	39	16	98	357	28	11	11	311	30	10	10	407	23	9	12	414	26	9	11	319	21	9	10	290	19	8	10	303	24	10	11	317	28	10	11	429	27	10	12	511	58	7	3-3-3
P08331	2',3'-cyclic-nucleotide 2'-phosphodiesterase precursor - [CN16_ECOLI]	P	40	18	95	444	34	15	16	236	18	9	10	290	19	9	10	353	25	12	12	346	19	9	10	301	20	10	12	278	13	7	8	257	15	8	10	214	12	6	7	647	71	6	3-3-3
P0AAY0	Uncharacterized protein ybiS precursor - [YBIS_ECO57]	P	41	8	32	150	26	5	5	97	19	4	4	57	9	3	3	131	34	6	6	100	19	3	4	97	27	4	5	99	20	3	5									306	33	6	3-3-1
P06610	Vitamin B12 transport periplasmic protein btuE - [BTUE_ECOLI]	P	42	5	31	111	26	3	4	66	18	2	2	99	28	3	4	229	42	5	7	120	25	3	4	39	19	2	2	68	18	2	2	99	27	3	4	96	18	2	2	183	20	5	3-3-3
P0ADU6	Protein ygiW precursor - [YGIW_ECO57]	P	42	5	20	69	35	3	3	134	33	4	6	50	14	2	2	75	22	3	3	72	14	2	2	70	14	2	2	47	22	2	2									130	14	5	3-3-1
P0AEU9	Chaperone protein skp precursor - [SKP_ECO57]	P	43	5	36	251	43	5	6	187	22	3	4	90	22	3	3	248	43	5	7	134	43	5	5	103	38	4	4	78	17	2	2	102	17	2	2	98	22	3	3	161	18	10	3-3-3
P18956	Gamma-glutamyltranspeptidase precursor - [GGT_ECOLI]	P	45	18	109	540	36	15	16	401	30	11	18	356	19	8	9	375	30	12	12	557	35	15	18	365	24	10	10	407	24	10	11	270	17	7	7	296	17	8	8	580	62	6	3-3-3
P0ABZ8	Chaperone surA precursor - [SURA_ECO57]	P	45	13	77	565	41	11	14	282	23	6	8	301	18	5	7	314	23	6	7	395	20	6	9	336	18	5	9	307	25	7	9	311	26	7	8	295	17	5	6	428	47	7	3-3-3
P0AEG5	Thiol:disulfide interchange protein dsbA precursor - [DSBA_ECO57]	P	47	8	40	238	47	7	7	160	34	5	5	179	33	5	5	124	25	3	4	224	33	5	5	194	33	5	5	194	23	3	3	116	24	3	3	121	24	3	3	208	23	6	3-3-3
P0AFK9	Spermidine/putrescine-binding periplasmic protein precursor - [POTD_ECOLI]	P	47	16	128	485	46	15	18	334	40	11	11	290	34	9	10	521	46	14	18	394	43	13	15	351	33	11	13	406	37	13	16	383	36	11	13	378	40	12	14	348	39	5	3-3-3
P76142	Uncharacterized protein yneA precursor - [YNEA_ECOLI]	P	48	9	53	339	48	9	11	189	35	6	7	61	6	2	2	199	36	6	7	167	28	6	7	114	26	5	5	175	31	6	6	97	21	4	4	123	25	4	4	340	37	8	3-3-3
P64598	Uncharacterized protein yraP precursor - [YRAP_ECO57]	P	48	6	21	196	39	5	6					99	13	2	2	256	41	5	5	102	22	3	3	75	13	2	2									111	22	3	3	191	20	9	2-3-1
P37329	Molybdate-binding periplasmic protein precursor - [MODA_ECOLI]	P	48	9	33	154	21	4	4	130	15	2	2					168	30	6	6	252	30	6	7	201	20	4	5	123	20	4	4	99	17	3	3	87	10	2	2	257	27	8	2-3-3
P0AGC4	Soluble lytic murein transglycosylase precursor - [SLT_ECO57]	P	51	22	97	321	19	10	10	328	29	11	12	380	25	14	14	303	17	9	9	525	23	13	17	289	22	11	12	148	10	6	6	148	11	6	6	244	16	9	11	645	73	9	3-3-3
P37902	Glutamate/aspartate periplasmic-binding protein precursor - [GLTI_ECOLI]	P	52	12	85	575	47	11	15	332	35	8	8	279	33	7	7	552	36	8	12	440	37	8	10	386	38	8	9	442	38	8	9	418	31	6	7	386	38	8	8	302	33	9	3-3-3
P0A9L3	FKBP-type 22 kDa peptidyl-prolyl cis-trans isomerase - [FKBB_ECOLI]	P	52	8	44	75	18	3	3	172	27	5	5	111	18	3	3	192	35	6	6	141	23	4	4	138	23	4	4	170	35	6	6	215	49	7	7	201	35	6	6	206	22	5	3-3-3
P0AFL5	Peptidyl-prolyl cis-trans isomerase A precursor - [PPIA_ECO57]	P	53	6	30	279	49	5	8	175	48	4	5					174	49	5	6	163	32	3	5	69	31	3	3					84	21	2	3					190	20	9	2-3-1
P16700	Thiosulfate-binding protein precursor - [CYSP_ECOLI]	P	53	16	81	400	27	9	11	424	34	9	12	170	16	5	5	360	46	13	15	201	21	7	8	205	25	7	7	227	23	7	7	200	22	6	6	307	28	8	10	338	38	8	3-3-3
P45523	FKBP-type peptidyl-prolyl cis-trans isomerase fkpA precursor - [FKBA_ECOLI]	P	54	13	118	622	46	10	14	484	50	11	16	359	48	10	12	432	44	9	14	530	54	13	18	446	44	10	13	352	38	8	9	324	44	9	10	347	48	10	12	270	29	8	3-3-3
P0AFH9	Osmotically-inducible protein Y precursor - [OSMY_ECOL6]	P	54	9	252	1359	52	8	31	1424	54	9	30	1133	52	8	28	1405	52	8	29	1585	52	8	40	1426	52	8	36	848	52	8	20	768	52	8	17	1088	52	8	21	201	21	7	3-3-3
P0AG81	sn-glycerol-3-phosphate-binding periplasmic protein ugpB precursor - [UGPB_ECO57]	P	54	17	104	390	34	10	12	323	37	12	12	399	39	12	13	280	31	10	11	581	46	14	16	357	28	10	11	372	31	11	11	269	28	9	9	227	29	9	9	438	48	7	3-3-3
P39099	Protease degQ precursor - [DEGQ_ECOLI]	P	54	20	158	817	40	14	20	799	43	15	18	485	37	12	15	818	43	15	18	488	45	14	16	460	38	12	15	647	41	15	18	723	43	16	19	767	40	15	19	455	47	6	3-3-3
P0AET0	Chaperone-like protein hdeA precursor - [HDEA_ECO57]	P	55	2	16	115	55	2	2	210	55	2	5	95	55	2	3	128	55	2	4	78	55	2	2																	110	12	5	3-2-0
A7ZKW9	Periplasmic trehalase precursor - [TREA_ECO24]	P	55	20	126	629	48	17	22	369	29	11	11	288	28	10	12	704	42	16	22	375	29	11	13	345	22	9	12	345	31	11	11	319	30	12	13	230	30	10	10	565	64	6	3-3-3
P09551	Lysine-arginine-ornithine-binding periplasmic protein precursor - [ARGT_ECOLI]	P	56	11	62	517	51	10	11	286	45	8	8	179	24	4	4	396	46	8	9	343	28	6	8	173	20	4	4	249	23	4	4	193	28	5	5	395	44	8	9	260	28	6	3-3-3
P0AEU2	Histidine-binding periplasmic protein precursor - [HISJ_ECO57]	P	56	10	59	268	41	7	8	123	22	4	5	159	26	5	5	328	44	8	10	308	48	9	10	130	22	4	4	235	33	6	7	174	26	5	5	191	26	5	5	260	28	6	3-3-3
P0AG82	Phosphate-binding protein pstS precursor - [PSTS_ECOLI]	P	62	19	155	782	58	17	19	412	44	12	12	594	54	15	17	651	53	15	17	654	56	16	19	663	54	15	17	707	56	16	19	615	53	15	17	662	54	15	18	346	37	8	3-3-3
P0ADV7	Protein yrbC precursor - [YRBC_ECOLI]	P	63	12	100	382	54	9	11	287	54	10	11	239	46	7	8	325	60	11	13	347	54	10	13	248	37	8	11	283	60	10	12	250	43	8	10	274	47	9	11	211	24	9	3-3-3
P00805	L-asparaginase 2 precursor - [ASPG2_ECOLI]	P	63	17	130	1000	63	17	26	507	42	13	13	729	50	14	19	475	41	10	10	585	48	14	15	461	35	10	12	321	41	10	10	343	39	10	10	549	52	13	15	348	37	6	3-3-3
P30859	Arginine-binding periplasmic protein 1 precursor - [ARTI_ECOLI]	P	65	14	104	474	51	11	14	352	63	12	14	169	39	7	8	365	55	12	15	311	46	9	11	270	42	8	10	293	35	7	11	184	42	8	9	323	42	8	12	243	27	6	3-3-3
P0AEE1	Protein dcrB precursor - [DCRB_ECOLI]	P	66	8	30	161	52	6	6					85	32	4	4	196	66	8	8	92	40	5	5	79	36	4	4	43	18	3	3									185	20	5	2-3-1
P0AEQ5	Glutamine-binding periplasmic protein precursor - [GLNH_ECO57]	P	69	16	118	619	63	14	19	546	60	11	14	371	50	10	10	615	60	12	18	413	58	12	14	330	46	9	11	377	37	8	11	338	53	11	12	324	45	9	9	248	27	9	3-3-3
P45955	Uncharacterized protein ybgF precursor - [YBGF_ECOLI]	P	70	10	34	322	40	6	6	192	13	2	2	151	16	3	3	400	52	6	8	272	43	6	6	160	13	2	2	208	17	2	3	161	17	2	2	138	17	2	2	263	28	9	3-3-3
P02925	D-ribose-binding periplasmic protein precursor - [RBSB_ECOLI]	P	70	15	172	912	70	15	24	946	70	14	22	731	68	12	19	969	70	15	24	677	60	13	18	764	55	11	18	667	51	11	16	613	51	11	16	606	51	10	15	296	31	7	3-3-3
P0AEE6	D-galactose-binding periplasmic protein precursor - [DGAL_ECOL6]	P	72	21	223	1068	68	19	29	881	62	16	23	831	58	15	22	992	65	18	28	860	58	16	25	1013	58	16	27	1131	62	16	26	940	62	16	22	846	63	16	21	332	36	6	3-3-3
P19926	Glucose-1-phosphatase precursor - [AGP_ECOLI]	P	73	19	121	728	63	17	22	595	68	15	19	364	37	10	11	524	45	13	14	491	53	15	16	351	25	8	9	399	37	10	11	342	32	9	9	339	32	9	10	413	46	6	3-3-3
P23847	Periplasmic dipeptide transport protein precursor - [DPPA_ECOLI]	P	75	28	297	1112	70	27	40	1649	55	23	51	744	40	17	23	1085	56	20	33	1037	48	20	32	891	51	20	29	1088	46	19	29	887	55	22	29	1063	57	22	31	535	60	7	3-3-3
P33362	Uncharacterized protein yehZ precursor - [YEHZ_ECOLI]	P	76	13	71	408	60	10	14	259	34	6	7	255	33	7	8	285	39	6	8	315	39	7	8	294	39	7	7	259	21	5	6	346	24	5	8	210	18	4	5	305	33	6	3-3-3
P76108	Putative ABC transporter periplasmic-binding protein ydcS precursor - [YDCS_ECOLI]	P	76	21	163	822	68	18	20	672	50	13	15	596	46	12	15	834	73	20	24	774	60	16	24	620	44	11	16	669	57	15	19	561	48	12	15	606	44	12	15	381	42	7	3-3-3
P0A864	Thiol peroxidase - [TPX_ECO57]	P	79	7	67	351	58	6	8	322	48	5	6	256	68	6	8	356	68	6	9	302	79	7	8	208	48	5	6	293	68	6	7	349	48	5	7	374	68	6	8	168	18	5	3-3-3
P23843	Periplasmic oligopeptide-binding protein precursor - [OPPA_ECOLI]	P	80	36	699	2861	78	32	85	2788	75	33	93	2573	73	32	85	2334	71	30	76	1965	67	28	62	2109	70	28	71	2457	71	29	81	2292	71	29	74	2186	73	33	72	543	61	6	3-3-3

P0AEN0	Cystine-binding periplasmic protein precursor - [FLIY_ECOL6]	P	83	24	181	920	77	21	27	672	61	16	21	390	40	10	11	860	73	18	24	680	71	19	22	671	66	17	19	702	71	18	20	685	68	17	19	682	62	15	18	266	29	7	3-3-3
P0AEY0	Maltose-binding periplasmic protein precursor - [MALE_ECO57]	P	83	25	267	1234	74	21	35	1467	80	23	42	813	62	18	25	1260	71	20	32	919	76	23	30	613	67	18	23	888	55	16	25	868	65	18	25	1020	62	18	30	396	43	6	3-3-3
P0ADS7	Uncharacterized protein yggE - [YGGE_ECO57]	P, IM	39	11	60	286	39	9	10	210	27	6	7	246	22	6	7	240	24	6	7	105	15	4	4	219	22	6	7	183	16	4	5	216	24	6	7	223	19	5	6	246	27	7	3-3-3
A7ZJC2	Protein tolB precursor - [TOLB_ECO24]	P, IM	46	12	70	356	34	8	10	496	31	7	12	151	13	4	4	315	33	9	10	280	26	8	8	189	15	5	5	231	13	4	5	297	25	7	8	302	22	7	8	430	46	8	3-3-3
P64605	Uncharacterized protein yrbD precursor - [YRBD_ECO57]	P, IM	50	8	32	134	42	6	6	43	25	2	2	46	17	2	3	134	25	4	5	51	22	3	3	94	27	4	5	42	27	3	3	106	32	5	5					183	20	5	3-3-2
P08506	D-alanyl-D-alanine carboxypeptidase dacC precursor - [DACC_ECOLI]	P, IM	53	16	86	453	47	13	15	105	20	5	5	282	36	9	9	498	51	15	15	348	35	10	10	307	36	9	10	176	27	7	7	384	38	10	10	107	21	5	5	400	44	8	3-3-3
P0C0V1	Periplasmic serine endoprotease DegP - [DEGP_ECO57]	P, IM	64	22	230	1067	54	18	27	1087	51	18	26	842	46	17	21	1273	49	19	27	1377	48	18	32	1057	44	16	28	893	45	16	21	1001	50	19	25	946	50	18	23	474	49	9	3-3-3
P77804	Protein ydgA precursor - [YDGA_ECOLI]	P, IM	69	24	174	866	69	24	27	508	35	12	14	483	40	15	16	695	61	20	23	649	46	17	21	527	37	14	16	374	29	12	14	751	65	21	27	470	47	15	16	502	55	5	3-3-3
A7ZTU5	ATP synthase gamma chain - [ATPG_ECO24]	IM	34	8	32	221	18	4	4	52	7	2	2	63	7	2	2	225	34	7	7	131	23	4	4	62	9	3	3	117	10	3	3	127	20	4	4	110	10	3	3	287	32	9	3-3-3
P64452	Uncharacterized lipoprotein ydcL precursor - [YDCL_ECO57]	IM	35	5	21	70	23	3	3	71	11	2	2					93	19	3	3	87	11	2	2					65	11	2	2	99	16	3	4	145	24	4	5	222	24	8	2-2-3
P69807	Mannose permease IID component - [PTND_ECO57]	IM	36	7	25	202	30	5	6	102	14	2	3	53	8	2	2	214	19	4	6	91	14	3	3									117	13	3	3	95	9	2	2	286	31	9	3-2-2
P0AC43	Succinate dehydrogenase flavoprotein subunit - [DHSA_ECO57]	IM	38	16	72	406	26	11	11	257	11	5	6	293	12	6	6	536	34	14	15	225	10	5	5	281	14	7	7	255	11	6	6	486	23	10	10	236	12	6	6	588	64	6	3-3-3
P0ABB9	Magnesium-transporting ATPase, P-type 1 - [ATMA_ECO57]	IM	38	25	184	985	37	23	32	519	29	17	17	599	22	13	18	981	33	21	30	569	26	17	20	680	18	13	20	355	17	11	11	670	31	19	23	358	21	13	13	898	99	6	3-3-3
P77737	Oligopeptide transport ATP-binding protein oppF - [OPPF_ECOLI]	IM	40	9	53	286	27	7	9	182	14	3	5	166	26	6	7	317	36	8	9	136	17	4	4	191	18	5	6	141	17	4	5	167	24	6	6	78	6	2	2	334	37	8	3-3-3
P33599	NADH-quinone oxidoreductase subunit C/D - [NUOCD_ECOLI]	IM	41	18	61	404	25	11	12	125	10	4	4	102	5	3	3	311	24	11	11	96	7	4	4	146	12	7	7	187	10	6	6	286	16	9	9	140	10	5	5	600	69	6	3-3-3
P0ABJ2	Ubiquinol oxidase subunit 2 precursor - [CYOA_ECOL6]	IM	42	7	41	250	37	6	8	75	26	3	4	153	13	3	3	206	37	6	6	151	9	2	3	148	26	4	4	109	13	2	2	206	29	5	7	78	22	3	4	315	35	7	3-3-3
P0ABI6	Magnesium transport protein corA - [CORA_ECO57]	IM	43	7	17	222	43	7	7	37	13	2	2					162	22	4	4	74	10	2	2						0			63	10	2	2					316	37	5	2-2-1
P0ADQ9	Uncharacterized protein ygaM - [YGAM_ECO57]	IM	45	3	48	141	33	2	4	407	33	2	11	62	33	2	2	113	33	2	4	90	33	2	3	58	33	2	2	77	33	2	3	345	33	2	12	260	45	3	7	113	12	8	3-3-3
P75818	Uncharacterized lipoprotein ybjP precursor - [YBJP_ECOLI]	IM	51	6	42	297	44	5	7	249	43	5	7	259	36	4	5	294	36	4	5	307	43	5	7	83	12	2	2	51	16	2	2	42	14	2	2	152	25	3	5	171	19	7	3-3-3
P0ABA2	ATP synthase B chain - [ATPF_ECO57]	IM	54	9	42	279	46	8	9	200	32	5	5	220	29	4	4	255	42	6	6	214	32	5	5	173	29	4	4	91	15	2	2	125	29	4	4	118	21	3	3	156	17	6	3-3-3
P0ADA6	Uncharacterized lipoprotein yajG precursor - [YAJG_ECOL6]	IM	55	6	23	156	43	4	4	169	35	3	5	51	21	2	2	112	18	3	3					80	15	2	2	82	12	2	2	95	12	2	2	121	18	3	3	192	21	9	3-2-3
P28635	D-methionine-binding lipoprotein metQ precursor - [METQ_ECOLI]	IM	57	10	69	489	47	8	10	132	23	4	4	282	38	6	8	409	45	8	10	258	31	5	6	142	28	5	5	206	35	5	6	353	47	8	9	326	54	9	11	271	29	5	3-3-3
P07014	Succinate dehydrogenase iron-sulfur subunit - [DHSB_ECOLI]	IM	58	12	55	393	42	8	9	196	16	3	4	70	11	2	2	456	55	11	13	196	30	6	6	71	11	2	2	131	16	3	3	423	52	11	12	223	22	4	4	238	27	7	3-3-3
P0AE07	Acriflavine resistance protein A precursor - [ACRA_ECO57]	IM	59	15	68	381	47	10	13	63	6	2	2	122	19	5	6	415	32	10	13	138	18	5	6	182	28	8	9	64	14	4	5	178	29	7	10	115	17	4	4	397	42	8	3-3-3
P0ADB2	Osmotically-inducible lipoprotein E precursor - [OSME_ECO57]	IM	67	5	36	244	67	5	7	249	67	5	7	127	38	4	4	229	67	5	7	226	67	5	6	81	20	3	3									79	28	2	2	112	12	8	3-3-1
A7ZTU6	ATP synthase subunit alpha - [ATPA_ECO24]	IM	70	27	205	1073	50	18	25	846	58	21	26	639	30	14	17	1101	58	22	31	667	35	13	18	667	35	13	15	720	46	18	23	909	55	20	26	905	45	18	24	513	55	6	3-3-3
P0AEH7	Protein elaB - [ELAB_ECO57]	IM	75	8	270	1126	65	5	40	1516	75	8	55	573	74	6	27	787	65	5	30	481	65	5	22	325	64	4	11	564	74	6	24	511	66	5	19	1054	74	6	42	101	11	6	3-3-3
A7ZTU4	ATP synthase subunit beta - [ATPB_ECO24]	IM	78	26	226	1116	66	21	31	853	47	14	19	761	43	14	18	1088	62	19	30	825	48	15	20	931	49	15	25	878	47	15	25	1084	59	20	30	1005	60	18	28	460	50	5	3-3-3
P64583	Uncharacterized protein yqjD - [YQJD_ECO57]	IM	80	9	162	596	74	8	18	614	80	8	23	470	74	7	14	576	74	7	20	692	74	7	20	482	64	6	12	739	74	8	22	465	73	6	14	591	74	8	19	101	11	9	3-3-3
P77717	Uncharacterized lipoprotein ybaY precursor - [YBAY_ECOLI]	IM	81	6	149	705	57	5	25	385	57	5	9	350	47	4	12	887	47	4	24	322	81	6	9	334	47	4	10	472	57	5	15	630	57	5	21	879	57	5	24	190	19	8	3-3-3
P0AFM7	Phage shock protein A - [PSPA_ECO57]	IM, C	37	7	24		0			148	18	3	3	152	18	4	4					55	8	2	2	77	14	3	3	83	17	3	3	124	24	4	4	142	24	4	5	222	25	5	2-2-3
P09373	Formate acetyltransferase 1 - [PFLB_ECOLI]	IM, C	38	18	75	349	23	11	12	64	3	2	2	134	11	5	5	127	8	4	4	206	13	7	8	174	9	6	6	407	23	11	13	498	25	12	16	243	18	9	9	760	85	6	3-3-3
P21367	Uncharacterized protein ycaC - [YCAC_ECOLI]	IM, C	42	6	60	267	42	6	8	314	42	6	10	226	42	6	6	228	42	6	8	141	30	4	4	115	28	3	3	176	42	5	5	236	42	6	7	303	42	6	9	208	23	5	3-3-3
P0AC49	Fumarate reductase iron-sulfur subunit - [FRDB_ECO57]	IM, C	43	6	30	195	29	4	4	63	17	2	2	77	20	4	4	98	20	4	4	101	20	4	4	82	16	3	3	45	11	2	2	94	29	4	4	59	16	3	3	244	27	7	3-3-3
Q8XDS0	Aspartate ammonia-lyase - [ASPA_ECO57]	IM, C	45	17	113	394	31	11	12	398	32	11	12	287	24	9	11	507	29	10	13	420	26	9	12	375	31	12	14	468	37	14	15	417	25	9	11	494	30	11	13	478	52	5	3-3-3
P0A9Q8	Aldehyde-alcohol dehydrogenase [Includes: Alcohol dehydrogenase - [ADHE_ECO57]	IM, C	47	25	128	828	36	19	21	463	20	13	14	615	26	15	17	570	23	15	16	660	21	12	15	715	23	14	18	505	14	9	12	266	11	7	7	323	12	8	8	891	96	7	3-3-3
P0AG32	Transcription termination factor rho - [RHO_ECO57]	IM, C	50	17	156	548	37	11	17	907	42	13	22	833	46	16	24	415	32	9	12	496	39	13	15	901	46	16	26	302	23	8	10	450	34	12	14	545	36	12	16	419	47	7	3-3-3
A7ZTH2	ADP-L-glycero-D-manno-heptose-6-epimerase - [HLDD_ECO24]	IM, C	53	14	110	567	47	12	14	321	35	9	9	379	35	9	10	447	42	11	12	398	37	10	11	372	27	7	8	573	48	13	15	615	50	13	16	563	45	12	15	310	35	5	3-3-3
P0ACG0	DNA-binding protein H-NS - [HNS_ECO57]	IM, C	55	9	68	116	40	4	4	344	55	7	11	336	46	7	11	357	46	7	9	400	55	7	10	386	46	6	12	90	30	4	4	124	29	3	4	65	23	2	3	137	16	5	3-3-3
P0AFG9	Pyruvate dehydrogenase E1 component - [ODP1_ECO57]	IM, C	57	41	206	590	25	17	20	1207	46	31	36	1041	41	28	35	628	24	15	18	1062	39	26	34	1226	35	26	33	300	9	7	9	329	11	8	9	424	16	11	12	887	100	6	3-3-3
A7ZKA9	Flavoprotein wrbA - [WRBA_ECO24]	IM, C	58	8	74	293	46	6	6	280	52	7	8	315	46	6	7	364	53	7	9	334	51	7	8	277	45	6	7	321	58	8	10	437	58	8	10	361	51	7	9	198	21	6	3-3-3
A7ZVS7	Purine nucleoside phosphorylase deoD-type - [DEOD_ECO24]	IM, C	62	11	68	280	32	7	10	243	43	8	9	168	19	4	4	254	32	7	8	172	22	5	6	151	21	5	5	203	40	8	9	258	36	7	9	223	35	7	8	239	26	6	3-3-3
P12758	Uridine phosphorylase - [UDP_ECOLI]	IM, C	64	9	74	223	51	7	7	170	43	5	5	184	36	5	6	248	56	7	8	171	47	5	6	173	38	6	7	361	53	8	11	404	60	8	13	338	60	8	11	253	27	6	3-3-3
P0AEK5	Enoyl-[acyl-carrier-protein] reductase [NADH] - [FABI_ECO57]	IM, C	67	11	57	131	13	2	4	128	36	5	5	95	10	2	3	188	35	6	7	86	12	3	3	67	8	2	2	403	48	9	11	371	40	7	11	404	45	8	11	262	28	6	3-3-3
A7ZHA4	Chaperone protein dnaK - [DNAK_ECO24]	IM, C	69	31	265	845	49	20	27	721	50	19	23	694	38	16	21	743	47	18	23	742	41	16	20	1022	47	19	26	1458	58	25	41	1507	68	28	42	1584	62	29	42	638	69	5	3-3-3
P0AD62	Pyruvate kinase I - [KPYK1_ECO57]	IM, C	70	30	286	1087	57	23	28	927	51	20	25	966	51	18	27	1085	57	24	32	1027	47	20	28	1071	53	20	30	1467	64	27	41	1498	66	28	39	1223	61	25	36	470	51	6	3-3-3
P0AE10	Alkyl hydroperoxide reductase subunit C - [AHPC_ECO57]	IM, C	73	11	107	445	50	7	12	415	50	7	13	307	42	6	9	430	50	7	12	355	64	8	12	363	36	5	9	384	45	8	13	376	50	7	11	472	66	9	16	187	21	5	3-3-3
A7ZSD0	Malate dehydrogenase - [MDH_ECO24]	IM, C	75	17	195	1011	69	13	19	986	72	16	24	717	68	14	18	1079	67	14	25	634	63	13	18	782	68	14	20	799	74	15	21	1170	75	16	28	783	70	15	22	312	32	6	3-3-3
P69909	Glutamate decarboxylase alpha - [DCEA_ECOL6]	IM, C	78	25	323					2082	69	21	59	1854	62	22	61													2188	57	21	65	2239	69	23	66	2372	69	23	72	466	53	5	2-0-3

P69911	Glutamate decarboxylase beta - [DCEB_ECO57]	IM, C	78	25	247	2329	66	22	60									2616	76	23	76	1563	62	21	50	1828	55	21	61													466	53	5	1-3-0
P0AGD5	Superoxide dismutase [Fe] - [SODF_ECO57]	IM, C	80	9	70	258	63	8	10	255	58	7	9	163	24	4	5	354	73	8	11	171	42	5	6	118	19	3	4	249	28	5	7	257	45	7	8	256	63	8	10	193	21	6	3-3-3
A7ZTR3	Tryptophanase - [TNAA_ECO24]	IM, C	84	29	522	1606	80	27	46	2352	76	25	69	1505	79	26	43	2599	81	27	71	1723	73	23	51	1317	68	24	47	###	76	28	67	2208	75	27	66	1976	73	27	62	471	53	6	3-3-3
A7ZPU1	Uracil phosphoribosyltransferase - [UPP_ECO24]	IM, C	85	12	86	358	78	10	13	236	48	6	6	326	48	7	9	361	48	7	9	292	40	6	9	343	48	7	10	384	52	8	10	383	51	7	10	306	48	7	10	208	23	5	3-3-3
A7ZSI4	DNA-directed RNA polymerase subunit alpha - [RPOA_ECO24]	IM, C	86	22	172	289	40	10	12	657	47	11	20	399	50	12	16	512	43	10	18	386	43	10	15	468	46	12	19	538	47	13	18	656	55	14	22	954	69	19	32	329	36	5	3-3-3
P0ABD4	Bacterioferritin - [BFR_ECOL6]	IM, C	87	17	994	3715	84	13	119	7608	87	17	263	3358	84	13	121	3124	84	13	110	3375	84	13	119	2428	84	12	81	2045	84	13	66	1673	84	13	52	1987	84	13	63	158	18	5	3-3-3
P77454	Glutaminase 1 - [GLSA1_ECOLI]	ND	34	4	11					69	25	2	3																	63	9	2	3	63	19	3	3	46	13	2	2	310	33	5	1-0-3
P0ADT1	Uncharacterized protein yggN - [YGGN_ECO57]	ND	34	5	14	73	15	2	2									57	15	2	2	126	21	3	4	90	23	4	4	66	10	2	2									239	26	9	1-3-1
P0AFM5	Phosphate starvation-inducible protein psiF precursor - [PSIF_ECOL6]	ND	40	4	15					148	32	3	4	116	40	4	4					184	40	4	5	36	19	2	2													106	12	10	2-2-0
A7ZHQ5	UPF0325 protein yaeH - [YAEH_ECO24]	ND	44	5	11	137	37	4	4	93	19	2	2					52	16	2	2	109	27	3	3																	128	15	7	2-2-0
Q46851	Uncharacterized protein yghZ - [YGHZ_ECOLI]	ND	44	9	43	81	13	3	3	168	26	4	5	92	13	4	4	81	18	3	3	217	14	4	5	188	11	3	4	165	21	5	5	220	28	5	7	190	31	6	7	346	39	7	3-3-3
P0C8J6	Tagatose-1,6-bisphosphate aldolase gatY - [GATY_ECOLI]	ND	44	12	66	167	26	5	6	200	26	6	8	103	26	5	5	229	34	9	9	129	31	7	7	70	11	4	4	212	36	8	9	243	25	8	10	180	31	7	8	284	31	6	3-3-3
P75691	Zinc-type alcohol dehydrogenase-like protein yahK - [YAHK_ECOLI]	ND	52	14	81	243	32	9	9	338	39	10	13	209	25	7	7	299	34	9	10	293	34	9	11	184	23	7	7	239	30	9	9	204	26	7	7	216	29	8	8	349	38	6	3-3-3
P0AAV6	Uncharacterized protein ybgS precursor - [YBGS_ECOLI]	ND	58	6	49	246	29	4	6	183	29	4	5	197	52	4	5	262	50	4	6	304	58	6	10	225	44	3	5	275	25	4	6	135	21	3	3	139	21	3	3	126	13	5	3-3-3
P76177	Protein ydgH precursor - [YDGH_ECOLI]	ND	60	17	119	686	51	14	17	449	44	11	11	580	49	12	16	504	45	12	16	596	52	14	17	711	47	12	17	264	21	5	5	420	32	7	9	394	35	8	11	314	34	9	3-3-3
P0ADE7	Uncharacterized protein ygaU - [YGAU_ECOL6]	ND	60	8	38	250	55	7	7	198	42	6	6	181	38	5	5	130	26	3	3	193	38	5	5	143	32	4	4	75	18	2	2	114	26	3	3	132	26	3	3	149	16	6	3-3-3
P0C8J8	Putative tagatose 6-phosphate kinase gatZ - [GATZ_ECOLI]	ND	68	16	112	544	60	13	18	729	54	13	21	277	45	10	11	424	49	11	15	148	20	5	5	100	5	2	2	288	32	8	11	448	43	11	17	269	36	9	12	420	47	6	3-3-3

Table S2. OMPs and PPs identified in C0 (30 ℃) and C1 (37 ℃) conditions without CHX-Dg by using the Progenesis LC-MS software. From the statistical parameters and criteria used to validate the identifications and the levels of protein expression (see the material and methods), no protein of this table shows a significant difference of expression between the two experimental conditions. Locations are assigned according UniProt and BioCyc databases: OM (Outer Membrane) and P (Periplasmic).

UniProt accession	Proteins	Location	Highest mean condition	q value	power	Max fold change	Peptides used for quantita- -tion
Identified proteins with sequence coverage score > 33,33%							
P23843	Periplasmic oligopeptide-binding protein precursor	P	C0	0.35	0.998	1.70	42
P0A9L3	FKBP-type 22 kDa peptidyl-prolyl cis-trans isomerase	P	C1	0.381	0.767	1.54	8
P0A915	Outer membrane protein W precursor	OM	C0	0.46	0.468	1.73	6
P0A913	Peptidoglycan-associated lipoprotein precursor	OM	C0	0.46	0.441	1.49	9
P0AEG5	Thiol:disulfide interchange protein dsbA precursor	P	C1	0.46	0.48	1.60	4
P0A864	Thiol peroxidase	P	C1	0.463	0.411	1.32	7
P45523	FKBP-type peptidyl-prolyl cis-trans isomerase fkpA precursor	P	C1	0.463	0.404	1.33	15
P02925	D-ribose-binding periplasmic protein precursor	P	C1	0.485	0.355	1.29	15
P64605	Uncharacterized protein yrbD precursor	P	C1	0.499	0.339	1.48	6
P0AFK9	Spermidine/putrescine-binding periplasmic protein precursor	P	C1	0.499	0.338	1.47	14
P02931	Outer membrane protein F precursor	OM	C0	0.515	0.275	1.09	19
P45955	Uncharacterized protein ybgF precursor	P	C1	0.515	0.287	1.37	8
P06610	Vitamin B12 transport periplasmic protein btuE	P	C1	0.515	0.287	1.57	3
P0AG81	sn-glycerol-3-phosphate-binding periplasmic protein ugpB precursor	P	C1	0.515	0.279	1.42	16
P39325	ABC transporter periplasmic-binding protein ytfQ precursor	P	C1	0.515	0.274	1.29	8
P37194	Outer membrane protein slp precursor	OM	C0	0.525	0.255	1.63	6
P0A902	Outer membrane lipoprotein blc precursor	OM	C0	0.526	0.244	1.93	7
A7ZJW0	Outer-membrane lipoprotein carrier protein precursor	OM	C1	0.531	0.201	1.29	7
P18956	Gamma-glutamyltranspeptidase precursor	P	C1	0.531	0.224	1.44	14
P0C0V1	Protease do precursor	P	C1	0.531	0.221	1.56	26
P0AG82	Phosphate-binding protein pstS precursor	P	C1	0.531	0.22	1.49	20
P0AEE6	D-galactose-binding periplasmic protein precursor	P	C1	0.531	0.216	1.34	20
P0ADV7	Protein yrbC precursor	P	C1	0.531	0.212	1.27	9
P0A919	Outer membrane protein X precursor	OM	C0	0.538	0.179	1.35	8
P37329	Molybdate-binding periplasmic protein precursor	P	C1	0.538	0.185	1.41	7

P0AGD2	Superoxide dismutase [Cu-Zn] precursor	P	C1	0.538	0.18	1.67	4
P76142	Uncharacterized protein yneA precursor	P	C1	0.538	0.179	1.27	9
P76116	Uncharacterized protein yncE precursor	P	C1	0.538	0.176	1.37	4
P37902	Glutamate/aspartate periplasmic-binding protein precursor	P	C1	0.538	0.174	1.35	9
P02930	Outer membrane protein tolC precursor	OM	C1	0.546	0.163	1.24	17
P09551	Lysine-arginine-ornithine-binding periplasmic protein precursor	P	C1	0.546	0.163	1.29	9
P77804	Protein ydgA precursor	P	C1	0.556	0.152	1.38	25
P04949	Flagellin	OM	C1	0.569	0.143	1.45	31
P0A928	Nucleoside-specific channel-forming protein tsx precursor	OM	C0	0.574	0.127	1.22	6
P08506	D-alanyl-D-alanine carboxypeptidase dacC precursor	P	C1	0.574	0.138	1.38	13
P0ADS7	Uncharacterized protein yggE	P	C1	0.574	0.128	1.23	11
A7ZKW9	Periplasmic trehalase precursor	P	C1	0.574	0.127	1.22	19
P07024	Protein ushA precursor [Includes: UDP-sugar hydrolase	P	C1	0.574	0.121	1.13	12
A7ZKF2	Glucans biosynthesis protein G precursor	P	C1	0.581	0.114	1.26	13
P39187	Uncharacterized protein ytfJ precursor	P	C1	0.581	0.113	1.30	4
P0AEU9	Chaperone protein skp precursor	P	C1	0.581	0.113	1.48	5
P0ABZ8	Chaperone surA precursor	P	C1	0.588	0.109	1.16	11
P0AFH9	Osmotically-inducible protein Y precursor	P	C1	0.588	0.109	1.39	8
P06996	Outer membrane protein C precursor	OM	C1	0.59	0.105	1.14	8
P0AEU2	Histidine-binding periplasmic protein precursor	P	C1	0.594	0.0997	1.26	6
P0AEQ5	Glutamine-binding periplasmic protein precursor	P	C1	0.6	0.095	1.13	16
A7ZJC2	Protein tolB precursor	P	C1	0.604	0.0924	1.20	11
P0AEN0	Cystine-binding periplasmic protein precursor	P	C1	0.611	0.0898	1.14	18
P39180	Antigen 43 precursor	OM	C1	0.614	0.0886	1.16	30
P0AEE1	Protein dcrB precursor	P	C1	0.622	0.0862	1.23	7
P31133	Putrescine-binding periplasmic protein precursor	P	C1	0.637	0.0803	1.15	8
P30859	Arginine-binding periplasmic protein 1 precursor	P	C1	0.638	0.0799	1.10	12
P23847	Periplasmic dipeptide transport protein precursor	P	C0	0.642	0.0771	1.16	31
P39099	Protease degQ precursor	P	C1	0.643	0.0768	1.08	14
P0AFL5	Peptidyl-prolyl cis-trans isomerase A precursor	P	C0	0.643	0.0764	1.46	5
A7ZQM2	Enolase - Escherichia coli O9:H4 (strain HS)	OM	C1	0.648	0.0742	1.09	21
P09394	Glycerophosphoryl diester phosphodiesterase precursor	P	C1	0.662	0.0711	1.14	4
P75937	Flagellar hook protein flgE	OM	C1	0.671	0.068	1.07	10
P00805	L-asparaginase 2 precursor	P	C0	0.674	0.0674	1.27	16
P33362	Uncharacterized protein yehZ precursor	P	C0	0.674	0.0641	1.32	11
P02943	Maltoporin precursor	OM	C1	0.681	0.0625	1.04	14

P64598	Uncharacterized protein yraP precursor	P	C1	0.681	0.0622	1.10	6
P0AEY0	Maltose-binding periplasmic protein precursor	P	C1	0.682	0.0617	1.08	28
P19926	Glucose-1-phosphatase precursor	P	C1	0.696	0.0587	1.09	15
A7ZKY3	Outer-membrane lipoprotein lolB precursor	OM	C1	0.697	0.0554	1.03	7
P0AGC4	Soluble lytic murein transglycosylase precursor	P	C1	0.697	0.0562	1.14	16
P0A911	Outer membrane protein A precursor	OM	C1	0.7	0.0545	1.03	25
P76108	Putative ABC transporter periplasmic-binding protein ydcS precursor	P	C1	0.7	0.0546	1.05	20
P0AAY0	Uncharacterized protein ybiS precursor	P	C0	0.71	0.0529	1.09	3
P08331	2'.3'-cyclic-nucleotide 2'-phosphodiesterase precursor	P	C0	0.719	0.0515	1.12	9
P0ADU6	Protein ygiW precursor	P	C1	0.72	0.0513	1.08	2
P06129	Vitamin B12 transporter btuB precursor	OM	C0	0.725	0.051	1.03	14
P16700	Thiosulfate-binding protein precursor	P	C0	0.73	0.0504	1.05	14
P0A906	Outer membrane lipoprotein slyB precursor	OM	C1	0.734	0.0502	1.30	6
P69778	Major outer membrane lipoprotein precursor	OM	C0	0.734	0.0501	1.01	5
P10384	Long-chain fatty acid transport protein precursor	OM	C1	0.737	0.05	1.00	10
P0AET0	Chaperone-like protein hdeA precursor	P	C1	0.737	0.05	1.09	2
P09169	Protease 7 precursor	OM	C0	0.741	0.05	1.01	4
identified proteins with sequence coverage score 33,33 %							
P0AD96	Leu/Ile/Val-binding protein precursor	P	C1	0.46	0.603	2.37	2
P73128	Probable D.D-dipeptide-binding periplasmic protein ddpA precursor	P	C0	0.463	0.388	1.72	5
A1A968	Glutathione-binding protein gsiB precursor	P	C1	0.51	0.303	1.42	9
P36649	Blue copper oxidase cueO precursor	P	C0	0.51	0.299	1.73	2
P37387	D-xylose-binding periplasmic protein precursor	P	C1	0.526	0.241	1.38	8
P25894	Uncharacterized metalloprotease yggG	OM	C1	0.531	0.202	1.65	2
P03841	Maltose operon periplasmic protein precursor	P	C1	0.531	0.209	1.73	4
A7ZJ30	LPS-assembly lipoprotein rlpB precursor	OM	C1	0.538	0.176	1.31	4
P02924	L-arabinose-binding periplasmic protein precursor	P	C1	0.538	0.184	1.43	3
A7ZP31	Ecotin precursor - Escherichia coli O157:H7	P	C1	0.539	0.168	1.35	3
A1AB32	Glucans biosynthesis protein D precursor	P	C1	0.55	0.16	1.28	8
P40710	Lipoprotein nlpE precursor	OM	C1	0.563	0.146	1.36	2
P77348	Periplasmic murein peptide-binding protein precursor	P	C1	0.574	0.127	1.31	11
P58320	Thiol:disulfide interchange protein dsbG precursor	P	C1	0.574	0.125	2.11	2
P21338	Ribonuclease I precursor	P	C1	0.628	0.0839	1.46	4
P0AE22	Class B acid phosphatase precursor	P	C1	0.638	0.0792	1.18	3
P0AET3	Protein hdeB precursor	P	C0	0.651	0.0737	2.27	2
A7ZHR7	Outer membrane protein assembly factor yaeT precursor	OM	C1	0.671	0.0682	1.08	14
P23843	Periplasmic appA protein precursor	P	C0	0.696	0.0582	1.24	4

P0A908	MltA-interacting protein precursor	OM	C1	0.697	0.0566	1.06	5
P78067	Putative thiosulfate sulfurtransferase ynjE precursor	P	C1	0.697	0.0558	1.05	9
P37648	Protein yhjJ precursor	P	C0	0.697	0.0557	1.04	3
P0AG78	Sulfate-binding protein precursor	P	C1	0.712	0.0522	1.12	2
P76193	Uncharacterized protein ynhG precursor	P	C1	0.712	0.0523	1.11	5
P21170	Biosynthetic arginine decarboxylase	P	C1	0.733	0.0503	1.02	4

AIMS Press

© 2017 Laurent Coquet, et al., licensee AIMS Press. This is an open access article distributed under the terms of the Creative Commons Attribution License (http://creativecommons.org/licenses/by/4.0)
